# Striking Similarity in the Gene Expression Levels of Individual Myc Module Members among ESCs, EpiSCs, and Partial iPSCs

**DOI:** 10.1371/journal.pone.0083769

**Published:** 2013-12-26

**Authors:** Masataka Hirasaki, Keiko Hiraki-Kamon, Masayoshi Kamon, Ayumu Suzuki, Miyuki Katano, Masazumi Nishimoto, Akihiko Okuda

**Affiliations:** 1 Division of Developmental Biology, Research Center for Genomic Medicine, Saitama Medical University, Hidaka, Saitama, Japan; 2 Core Research for Evolutional Science and Technology (CREST), Japan Science and Technology Agency, Kawaguchi, Saitama, Japan; Kanazawa University, Japan

## Abstract

Predominant transcriptional subnetworks called Core, Myc, and PRC modules have been shown to participate in preservation of the pluripotency and self-renewality of embryonic stem cells (ESCs). Epiblast stem cells (EpiSCs) are another cell type that possesses pluripotency and self-renewality. However, the roles of these modules in EpiSCs have not been systematically examined to date. Here, we compared the average expression levels of Core, Myc, and PRC module genes between ESCs and EpiSCs. EpiSCs showed substantially higher and lower expression levels of PRC and Core module genes, respectively, compared with those in ESCs, while Myc module members showed almost equivalent levels of average gene expression. Subsequent analyses revealed that the similarity in gene expression levels of the Myc module between these two cell types was not just overall, but striking similarities were evident even when comparing the expression of individual genes. We also observed equivalent levels of similarity in the expression of individual Myc module genes between induced pluripotent stem cells (iPSCs) and partial iPSCs that are an unwanted byproduct generated during iPSC induction. Moreover, our data demonstrate that partial iPSCs depend on a high level of c-Myc expression for their self-renewal properties.

## Introduction

Embryonic stem cells (ESCs) derived from blastocysts are able to self-renew indefinitely and bear pluripotency, which is defined as the property enabling differentiation into any cell type of the entire body [1-3]. Because of these remarkable biological properties, ESCs are expected to be an unlimited source of functionally mature differentiated cells for therapeutic purposes, such as cardiomyocytes and pancreatic insulin-secreting β cells. Indeed, the first clinical trial was conducted in 2012 for ESC-derived cell transplantation into patients with optical diseases [4]. The self-renewality and pluripotency of ESCs are sustained by the combinatorial actions of numerous transcriptional factors including Oct3/4, Sox2, and Nanog [5-9]. In addition, intricate epigenetic controls [10-15] and various signaling pathways [16-19] are intermingled with this transcriptional network to establish the very sophisticated ESC status.

Recent comprehensive protein interaction and target gene assessment of each core pluripotency factor, polycomb complex factor, and Myc-related factor has provided a framework for the conceptual regulatory network that is crucial to support the mouse ESC status [20]. Three transcriptional subnetworks have been defined as Core, Myc, and PRC modules consisting of 111, 503, and 560 genes, respectively. Importantly, only few genes overlap between two different modules, and none of the genes are common among all three modules, indicating that the function of each module is independent. In mouse ESCs, most members of Core and Myc modules show high expression levels compared with those in differentiated derivatives, while most PRC module members show a contrasting expression pattern, suggesting that Core and Myc module members, but not PRC module members, actively participate in sustaining the ESC status.

Epiblast stem cells (EpiSCs) derived from the epiblast of postimplantation embryos (5.5–6.5 days postcoitum) also possess pluripotency and indefinite self-renewality [21-24], although the latter property is not as stable as that of ESCs. However, Core, Myc, and PRC module gene members have not been examined in the transcriptional network of EpiSCs.

Here, we conducted a detailed examination of the expression of Core, Myc, and PRC module genes in ESCs and EpiSCs. We found Core and PRC module gene expression is low and high in EpiSCs, respectively, compared with that in ESCs, while Myc module gene expression is equivalent between these two cell types. More importantly, the equivalence of Myc module gene expression is not just overall. Most of the Myc module genes show comparable expression levels in ESCs and EpiSCs. The same trend is found when comparing Myc module activity between induced pluripotent stem cells (iPSCs) and partial iPSCs that stray from the normal reprogramming route and become immortalized [25]. These observations indicate that Myc module members exert specific biological effects that are commonly important for ESCs/iPSCs, EpiSCs, and partial iPSCs. In consistent with this idea, our data demonstrate that exogenous supply of c-Myc expression is crucially involved in self-renewal property of partial iPSCs by positively regulating Myc module gene expression.

## Materials and Methods

### Ethics Statement

Preparation of mouse embryonic fibroblasts (MEFs) from transgenic mice bearing a *Nanog-green fluorescent protein* (*GFP*) reporter gene [26] was carried out in accordance with international and institutional guidelines. The protocol was approved by the Institutional Review Board for the Ethics of Animal Experiments of Saitama Medical University (permit number: 24G26). All surgeries were performed after sacrifice by cervical dislocation under anesthesia with diethyl ether inhalation.

### Generation and culture of partial iPSC clones and epiblast-like cells (EpiLCs)

To generate partial iPSC clones, MEFs bearing a *Nanog-green fluorescent protein* (*GFP*) reporter gene [26] were subjected to iPSC induction with retroviruses carrying Oct3/4, Sox2, Klf4, c-Myc, or rtTA. All cDNAs except for c-Myc were subcloned into pMXs vectors containing a Moloney murine leukemia virus (MMLV) long terminal repeat (LTR) [27]. c-Myc cDNA was linked to a tTA responsive element-containing promoter in the retroviral vector that also carried the *DsRed* gene under the control of the MMLV 5’ LTR. One hundred colonies generated at 3 or 4 weeks post-iPSC induction, which were positive for DsRed but negative for *Nanog* gene promoter-dependent GFP expression, were individually recovered and maintained on feeder cells as candidates for partial iPSCs. After elimination of clones that failed to propagate robustly and/or tended to spontaneously convert to genuine iPSCs, one clone (#55) was chosen based on efficient conversion to GFP-positive iPSCs by exposure to 2i (MAPK and GSK3β inhibitors) and leukemia inhibitory factor (LIF) [28]. Partial iPSCs were cultured in the presence of doxycycline (Dox) with standard mouse ESC medium containing fetal bovine serum and LIF on feeder cells unless indicated otherwise. CMT-1 ESCs cultured under the 2i condition [28] were induced to EpiLCs with activin A, basic fibroblast growth factor, and 1% knockout serum replacement as described by Hayashi et al. [29].

### Quantitative RT-PCR

RNA was recovered using Trizol reagent from EpiLCs, partial iPSCs, and iPSCs converted from partial iPSCs. Partial and genuine iPSCs were cultured under Dox-treated and untreated conditions. SYBR Green-based quantitative RT-PCR was performed using a StepOnePlus Real-time PCR System (Applied Biosystems). Primers used for the analyses are listed in Table S1. All samples were tested in triplicate and the results normalized to *GAPDH* expression levels.

### Western blotting and alkaline phosphatase staining

Alkaline phosphatase staining was conducted using a Leukocyte Alkaline phosphatase kit from Sigma. Western blot analyses were performed as described previously [30].

### Microarray data processing

Gene expression profiling data used for analyses were obtained from the Gene Expression Omnibus (GEO) database as follows. GSE30056 associated with Hayashi et al. [29] for EpiSCs and EpiLCs; GSE14012 associated with Sridharan et al. [31] for MEFs, iPSCs, and piPSCs; GSE34799 associated with Rugg-Gunn et al. [32] for ESCs and EpiSCs; GSE21222 associated with Hanna et al. [33] for human iPSCs in naïve and primed states; GSE11274 associated with Ko et al. [34] for germline stem cells (GSCs), germline-derived pluripotent stem cells (gPSCs), and neural stem cell (NSC)s; GSE31028 associated with Lien et al. [35] for quiescent and activated hair follicular stem cell (HFSC)s; GSE6506 associated with Chambers et al. [36] for long-term hematopoietic stem cell (LT-HSC)s, granulocytes, and B cells; GSE9954 associated with Thorrez et al. [37] for ovary, testis, bone marrow, placenta, adipose tissue, kidney, liver, pancreas, lung, brain, and heart; GSE19233 associated with Walker et al. [38] for bone marrow mesenchymal stem cell (MSC)s; GSE31150 associated with Pardo et al. [39] for pancreas. Microarray expression data were background subtracted and normalized by the robust multi-array analysis method [40] using R-package 2.8.1 with Bioconductor 2.6 [41]. Spotfire X.X. (TIBCO) was used to construct scatter plots.

## Results

### Comparison of gene expression of Core and Myc module members between ESCs and EpiSCs

**Figure 1:**
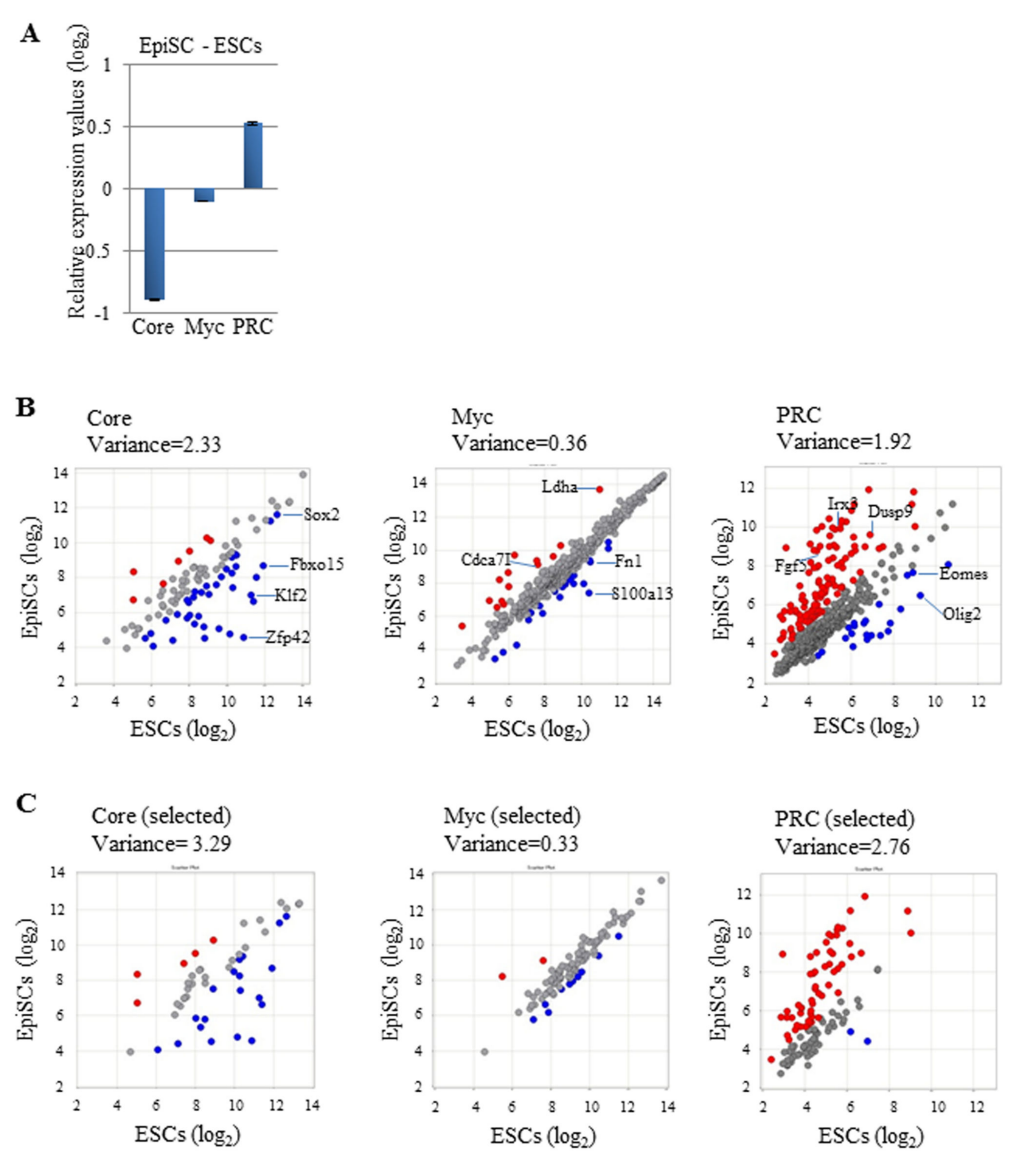
Comparison of the expression of Core and Myc module genes in EpiSCs and ESCs. (A) Average gene expression values (log_2_) of Core, Myc, and PRC module genes in EpiSCs using values from ESCs as references. Data from 99 Core, 426 Myc, and 474 PRC module genes deposited in GEO under GSE30056 were used for the analyses. Data from 12 Core (111 genes), 77 Myc (503 genes), and 86 PRC (560) module genes are not available in the deposited data sets. (B) Comparison of the expression of individual Core, Myc, and PRC module genes between ESCs and EpiSCs. Left, middle, and right scatter plots show the expression values of individual Core, Myc, and PRC module genes, respectively, in ESCs and EpiSCs. Red and blue spots indicate genes with expression levels that are higher or lower by more than 2-fold in EpiSCs compared with those in ESCs, respectively. Gene symbols corresponding to red and blue are listed in Table S2. The variance value was calculated and is shown for each scatter plot. (C) Left, middle, and right scatter plots show the expression values of the selected Core, Myc, and Core module genes (listed in Table S3), respectively, in ESCs and EpiSCs. Red and blue spots indicate as described in B. The variance value was calculated and is shown for each scatter plot.

To compare gene expression levels of Core, Myc, and PRC module members between ESCs and EpiSCs, we first used data deposited by Hayashi et al. [29] in NCBI GEO under GSE30056. The average gene expression levels of Core and PRC module genes were about 1.8-fold lower and 1.4-fold higher in EpiSCs than those in ESCs, respectively (Figure 1A). A lower expression value of the Core module in EpiSCs was expected because *Klf2*, *Fbox15*, and *Nanog*, all of which are members of the Core module, have previously shown very low expression in EpiSCs [22,23]. A higher expression value of the PRC module in EpiSCs may reflect the fact that EpiSCs correspond to cells in a more developmentally progressed embryonic stage than that of ESCs. Alternatively, difference in culture condition may cause this difference. Interestingly, our data demonstrated that EpiSCs and ESCs showed comparable gene expression of Myc module members, which was unexpected, because Myc expression has been shown to function negatively in self-renewal of human ESCs [42] that are more similar to mouse EpiSCs than mouse ESCs [21,22]. Next, we constructed scatter plots to compare the expression of individual Core module members (Figure 1B). As a result, 37 out of 99 genes (37.4%), including *Klf2* and *Fbox15*, were down-regulated by more than 2-fold in EpiSCs compared with that in ESCs, while only seven genes (0.71%) showed more than 2-fold higher expression in EpiSCs than that in ESCs (Table S2). The same scatter plot analyses were applied to compare the expression levels of individual Myc module members between ESCs and EpiSCs (Figure 1B). These analyses revealed that 92% (392 genes) of Myc module members showed comparable expression between ESCs and EpiSCs. Only 14 (3.3%) and 20 genes (4.7%) showed relatively higher and lower expression levels in ESCs, respectively (Table S2). For the PRC module, 113 (24%) and 22 (4.6%) genes out of 474 genes were up- and down-regulated in EpiSCs compared with those in ESCs, respectively. Not all Core and Myc module members showed higher expression in pluripotent cells including ESCs and iPSCs compared with that in differentiated somatic cells. Likewise, not all PRC module members showed lower expression in pluripotent cells compared with that in differentiated cells. In particular, many Myc module genes showed equally high expression in MEFs and iPSCs (Figure S1). Therefore, we considered that the genes with equivalent expression in all three cell types, i.e., ESCs, EpiSCs and MEFs, substantially contributed to the high similarity in gene expression of individual Myc module members between ESCs and EpiSCs. To eliminate this possibility, we selected Core and Myc module genes with expression levels that were increased by more than 2-fold in iPSCs compared with those in MEFs. For PRC module genes, we selected genes that showed contrasting expression patterns, i.e., higher expression in MEFs than that in iPSCs. We then compared the expression of genes that met this criterion (50, 98, and 115 genes in Core, Myc, and PRC module genes, respectively) (Table S3) in ESCs and EpiSCs. As shown in Figure 1C, even after this selection, we found strongly conserved expression profiles of Myc module genes in ESCs and EpiSCs, while Core and PRC module genes showed highly variable expression profiles. Gene set enrichment analyses (GSEAs) also demonstrated preferential expression of Core and PRC module genes in ESCs and EpiSCs, respectively, while equivalent expression levels of Myc module genes were found in these two cell types (Figure S2). We also performed the same set of analyses using the deposited data in GEO under GSE34799 [32] for gene expression in ESCs and EpiSCs. Subsequently, we obtained comparable results (Figure S3) to those shown in Figure 1 at least for Core and Myc module gene expression. However, unlike the results shown in Figure 1A, preferential expression of PRC module genes in EpiSCs was not evident in this data set. It is unclear why EpiSC lines generated in different laboratories showed different data for PRC module gene expression. One possibility is that this difference was caused by the different methods used to establish the EpiSCs. In Figure 1, the EpiSCs were derived from mouse post-implantation embryos, whereas those used in Figure S2 were generated *in vitro* from ESCs.

### Epiblast-like cells bear comparable Myc module gene expression

**Figure 2:**
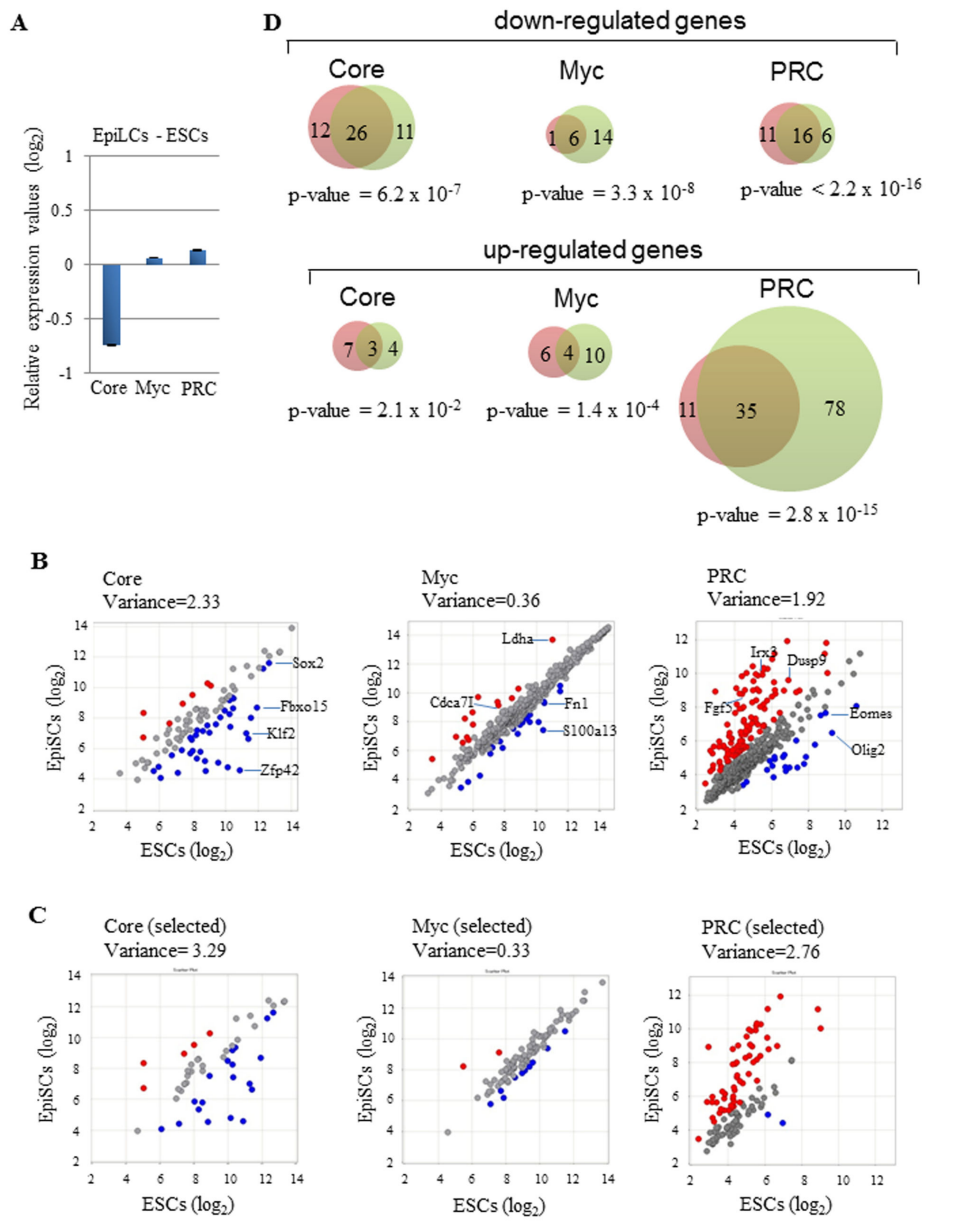
EpiLCs and EpiSCs show similar expression of Core, Myc, and PRC module genes. (A) Average gene expression values (log_2_) of Core, Myc, and PRC modules in EpiLCs were calculated using those in ESCs as references. The same GEO data (GSE30056) used in Figure 1 were used for the analyses. (B) Left, middle, and right scatter plots show the expression of individual Core, Myc, and PRC module genes, respectively, in ESCs and EpiLCs. The variance value was calculated and is shown for each scatter plot. Red and blue spots correspond to genes that show more than 2-fold higher or lower expression in EpiLCs compared with that in ESCs, respectively. Gene symbols corresponding to red and blue are listed in Table S4. (C) Scatter plots were constructed for the selected genes from Core (left), Myc (middle), and PRC (right) modules. The same sets of genes (Table S3) used in Figure 1C were used for the analyses. Red and blue spots indicate as described in B. (D) Venn diagrams demonstrating the relationship between genes with more than 2-fold higher or lower expression in EpiSCs (Figure 1B) and EpiLCs (B) compared with that in ESCs for Core, Myc, and PRC module genes. Numbers in pink and green circles indicate the number of genes which show differential expression levels specifically in EpiLCs and EpiSCs. The numbers in overlapping portions of two circles indicate the number of commonly up- or down-regulated genes in EpiSCs and EpiLCs compared with those in ESCs and the names of those genes are indicated with red letters in Table S2 and Table S4. Fisher’s Exact Test was conducted to calculate the p-values.

Because we obtained unexpected results for Myc module gene expression in EpiSCs, we performed further characterizations to gain more insight into this finding. EpiSCs are derived from the epiblast of postimplantation embryos. However, global gene expression analyses have revealed that there is significant dissimilarity between EpiSCs and *in vivo* epiblast cells. EpiLCs are generated from ESCs by exposure to activin A, basic fibroblast growth factor, and a low concentration of knockout serum replacement [29]. Principal component analyses and a high competency for primordial germ cell fate shown by Hayashi et al. [29] have demonstrated that EpiLCs represent a much more faithful *in vitro* counterpart of the epiblast of early mouse embryos than that of EpiSCs. Therefore, we determined whether EpiLCs also show prominent Myc module gene expression or whether high expression of Myc module genes is an adaptation of epiblast cells from postimplantation embryos for establishment as a cell line, i.e., EpiSCs. Figure 2A–C shows the results of analyses using all of the available Core, Myc, and PRC module genes and the selected genes shown in Figure 1C (see Table S4 for the list of differentially expressed genes in ESCs and EpiLCs). Comparison of Myc, Core, and PRC module gene expression between ESCs and EpiLCs gave rise to essentially the same results obtained by comparison between ESCs and EpiSCs, albeit PRC module gene expression in EpiLCs was not as high as that observed in EpiSCs. Thus, we concluded that high expression levels of Myc module members is not specific to artificially stabilized EpiSCs, but also occurs in more physiologically relevant EpiLCs. To confirm the relevance of the results obtained from comparisons of gene expression in ESCs and EpiSC/EpiLCs, we compared ubiquitous expression of housekeeping genes among these cell types. As shown in Figure S4A, seven out of eight housekeeping genes showed comparable expression among ESCs, EpiSCs, and EpiLCs, which validated that our normalization of the deposited gene expression data was conducted appropriately. Only the *pgk-1* gene showed higher expression in EpiSCs/EpiLCs compared with that in ESCs, although we do not know the physiological meaning of this finding at present. Since *pgk-1* gene is localized in X chromosome, we inquired whether the above result was something to do with X chromosome localization of the gene. However, we found that no such obvious trend was evident with the expression analyses of 6 other ubiquitously expressed X chromosome genes (Figure S5). Figure 2D shows the comparison between EpiSCs and EpiLCs for genes with differential expression in either EpiSCs or EpiLCs compared with that in ESCs (commonly up- or down-regulated genes in EpiSCs and EpiLCs compared with those in ESCs are indicated with red letters in Table S2 and Table S4). We found a strong overlap among Core module genes with lower expression in EpiSCs compared with that in ESCs and genes with lower expression levels in EpiLCs. This result suggests that these 26 genes corresponding to 26.3% of all Core module genes contribute at least in part to phenotypic differences between ESCs and EpiSCs/EpiLCs. We also found that there was a significant overlap of differentially expressed PRC module genes in ESCs and EpiSCs and those in ESCs and EpiLCs. However, we could not determine any obvious biological implication by examining the list of differentially expressed PRC module genes (Table S2 and Table S4). Although we also noted that there were some commonly up- or down-regulated Myc module genes in EpiSCs and EpiLCs compared with that in ESCs, these genes corresponded to only 1.4% and 0.9% of total Myc module genes, respectively. Therefore, it is likely that the finding of common activation of the majority of Myc module genes (92%) among ESCs, EpiSCs, and EpiLCs is more biologically relevant than identifying genes with differential expression levels in ESCs and EpiSCs/EpiLCs. However, we did not completely eliminate the possibility that the differentially expressed Myc module genes may contribute to characteristic differences between ESCs and EpiSCs/EpiLCs to some extent. 

To validate our computational data, we prepared EpiLCs to compare the expression of some differentially expressed Myc module genes by quantitative RT-PCR. These analyses revealed that all of the examined genes showed the expected changes in expression levels between ESCs and EpiLCs (Figure S6A and S6B). We also examined some of Myc module genes showing comparable expression levels between ESCs and EpiLCs, but higher compared to MEFs by quantitative RT-PCR and found that seven out of eight genes which were examined showed expected results. Only *Nc1* gene showed more than 2-fold lower expression level in EpiLCs compared to ESCs (Figure S6C), but we don’t know the physiological significance of this finding at present. Gene Ontology analyses revealed one particular term, the Brix domain, which was significantly enriched among down-regulated Myc module genes in either EpiSCs or EpiLCs (data not shown). Indeed, we found two (*Imp4* and *Ppan*) out of 20 down-regulated Myc module genes in EpiSCs compared with those in ESCs belonged to this category. Because these genes are involved in ribosomal RNA processing, it is possible that down-regulation of these two genes in EpiSCs may have a causal link to their slow proliferation rate compared with that of ESCs. In addition to the bioinformatics, it may be noteworthy that fibronectin 1 (Fn1) was identified as a down-regulated gene in EpiSCs and EpiLCs compared with that in ESCs, because this difference in expression may contribute to morphological differences between these cell types. It is also noteworthy that c-Myc gene by itself belongs to PRC module gene and 8 times higher expression in EpiLCs compared to ESCs (Table S4). However, higher c-Myc expression did not lead to elevation of average expression level of Myc module gene in EpiLCs. Since at least 6 genes (*Dmap1, E2F1, E2F4, Zfx, Max, N-Myc*) in addition to c-Myc participates in controlling expression of Myc module genes [20], we assume that only one member of modulators of Myc module gene expression is not enough to up-regulate overall expression level of Myc module genes. Consistent with this notion, we found none of other modulators of Myc module genes showed higher expression in EpiLCs compared to ESCs (Figure S7).

### Striking similarity of Myc module gene expression in naïve and primed human iPSCs

**Figure 3:**
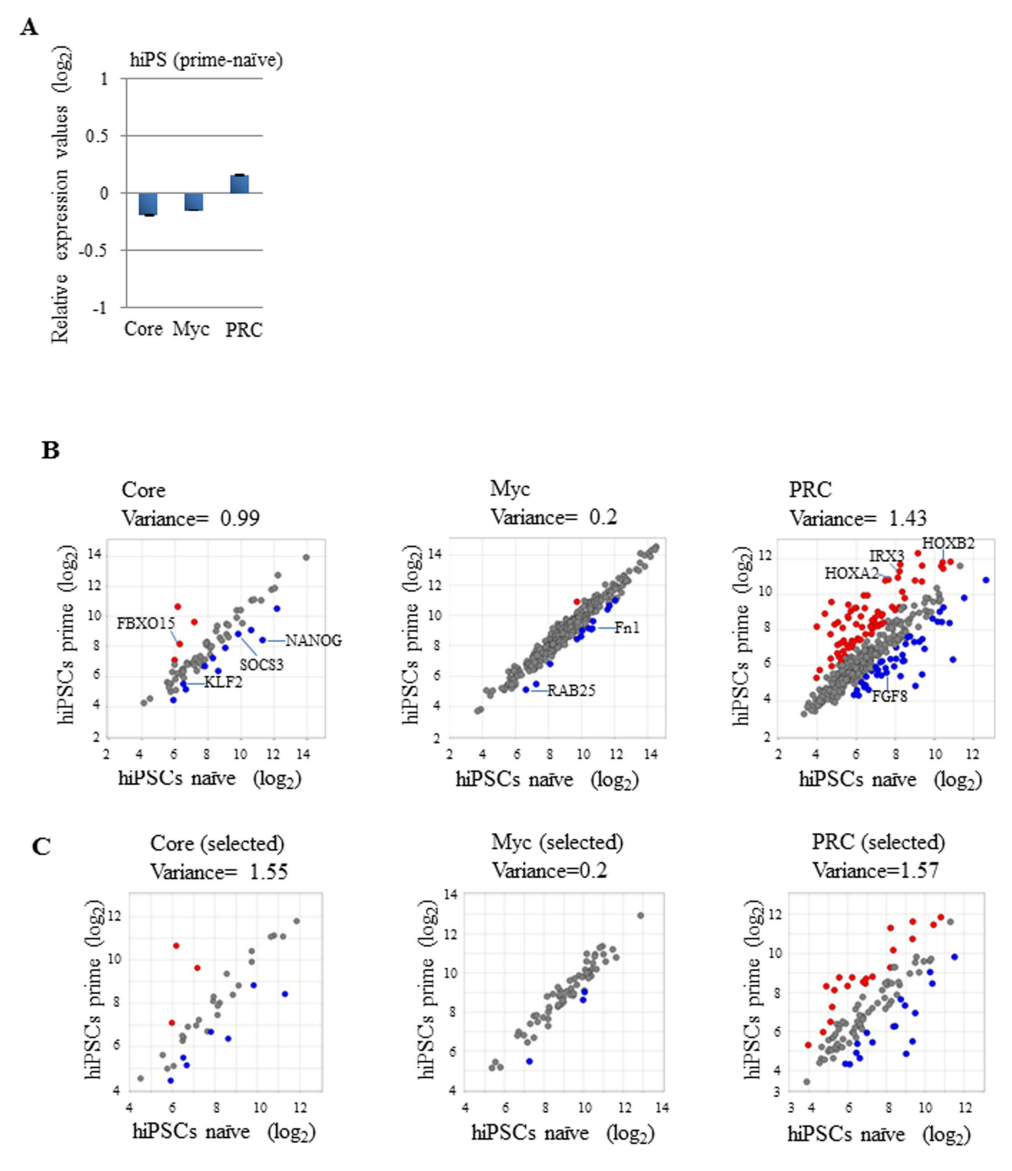
Most Myc module members maintain constant levels of expression in naïve and primed human iPSCs. (A) Average gene expression values (log_2_) of Core, Myc, and PRC module genes in primed human iPSCs using those in human iPSCs converted to a naïve state as references 33. Data from 69 Core, 321 Myc, and 423 PRC module genes deposited in GEO under GSE21222 were used for the analyses. Data from six Core, 34 Myc, and 28 PRC module genes are not available in the deposited data sets. (B) Comparison of the expression of individual Core, Myc, and PRC module genes between naïve and primed human iPSCs. Left, middle, and right scatter plots show the expression values of individual Core, Myc, and PRC module genes, respectively, in naïve and primed human iPSCs. Red and blue spots indicate genes with expression levels that are higher or lower by more than 2-fold in primed human iPSCs compared with those in naïve human iPSCs, respectively. Gene symbols corresponding to red and blue are listed in Table S5. The variance value was calculated and is shown for each scatter plot. (C) Scatter plot analyses of the selected genes from Core (left), Myc (middle), and PRC (right) modules. The same sets of genes (Table S3) used in Figure 1C were used for the analyses. The data lacked information for 15, 33, and 13 genes of the selected Core (50), Myc (98), and PRC (115) module genes, respectively. Red and blue spots indicate as described in B.

Although mouse and human ESCs are derived from pre-implantation blastocysts, there are significant characteristic differences between ESCs of these two species. iPSC technology has allowed generation of pluripotent cells using fully differentiated somatic cells as starting materials [43,44]. Human iPSCs are essentially the same as human ESCs in terms of their biological properties. Likewise, mouse iPSCs are equivalent to mouse ESCs. Therefore, human iPSCs bear the same levels of dissimilarity to mouse iPSCs as those observed in the comparison between human and mouse ESCs. Human ESCs/iPSCs are much more similar to mouse EpiSCs than mouse ESCs in many aspects such as required culture conditions, overall gene expression profile, and epigenetic modification status [21,22]. Therefore, it is generally considered that human ESCs fail to stabilize themselves in a mouse ESC-like naïve state, but proceed through embryogenesis in culture dishes spontaneously up to a stage equivalent to that of mouse EpiSCs. However, it is unknown whether human iPSCs are generated via a naïve state or directly reaches to EpiSC-like state during iPSC induction. Several groups have demonstrated that human ESCs/iPSCs can be maintained in a mouse ESC-like naïve state at least transiently by overexpression of several pluripotency factors including Oct3/4 and Klf4 [33,45]. Therefore, we compared gene expression of Core, Myc, and PRC modules between human iPSCs in a mouse ESC-like naïve state and those in a typical state (primed) with publicly available DNA microarray data deposited in NCBI GEO (GSE21222) [33]. As a result, we found that the difference in average expression of Myc module genes was equivalently small compared with the difference between mouse ESCs and EpiSCs (Figure 3A). However, the equivalently small difference was also evident in average expression of Core module genes between naïve and primed human iPSCs. This result was quite different from that obtained by the comparison between mouse ESCs and EpiSCs, suggesting that so-called “naïve” human ESCs/iPSCs do not represent a genuine human counterpart of mouse ESCs. We also found that primed human iPSCs showed more active PRC module gene expression than that in naïve cells. However, the difference was not as significant as that observed in the comparison between mouse ESCs and EpiSCs, but equivalently modest compared with that observed in the comparison between ESCs and EpiLCs. Next, we conducted scatter plot analyses as described in Figure 1B. As a result, although Core and PRC module genes showed highly variable expression patterns in naïve and primed human iPSCs, most Myc module genes showed comparable levels of expression in these two states (Figure 3B). We also conducted the same analyses with the selected genes used in Figure 1C and reached the same conclusion (Figure 3C). Similar to mouse ESCs/EpiSCs, most Myc module genes in human iPSCs do not show significantly different expression levels between their naïve and primed states. Recently, Gafni et al. [46] have successfully generated human ground state naïve pluripotent cells that are independent of expression of exogenous reprogramming factors. Therefore, we have conducted the same analyses with these newly established human cell lines and found very similar trend observed with exogenous reprogramming factor-dependent mouse ESC-like human ESCs/iPSCs (Figure S8).

### Core, Myc, and PRC module gene expression in various tissues and somatic stem cells

**Figure 4:**
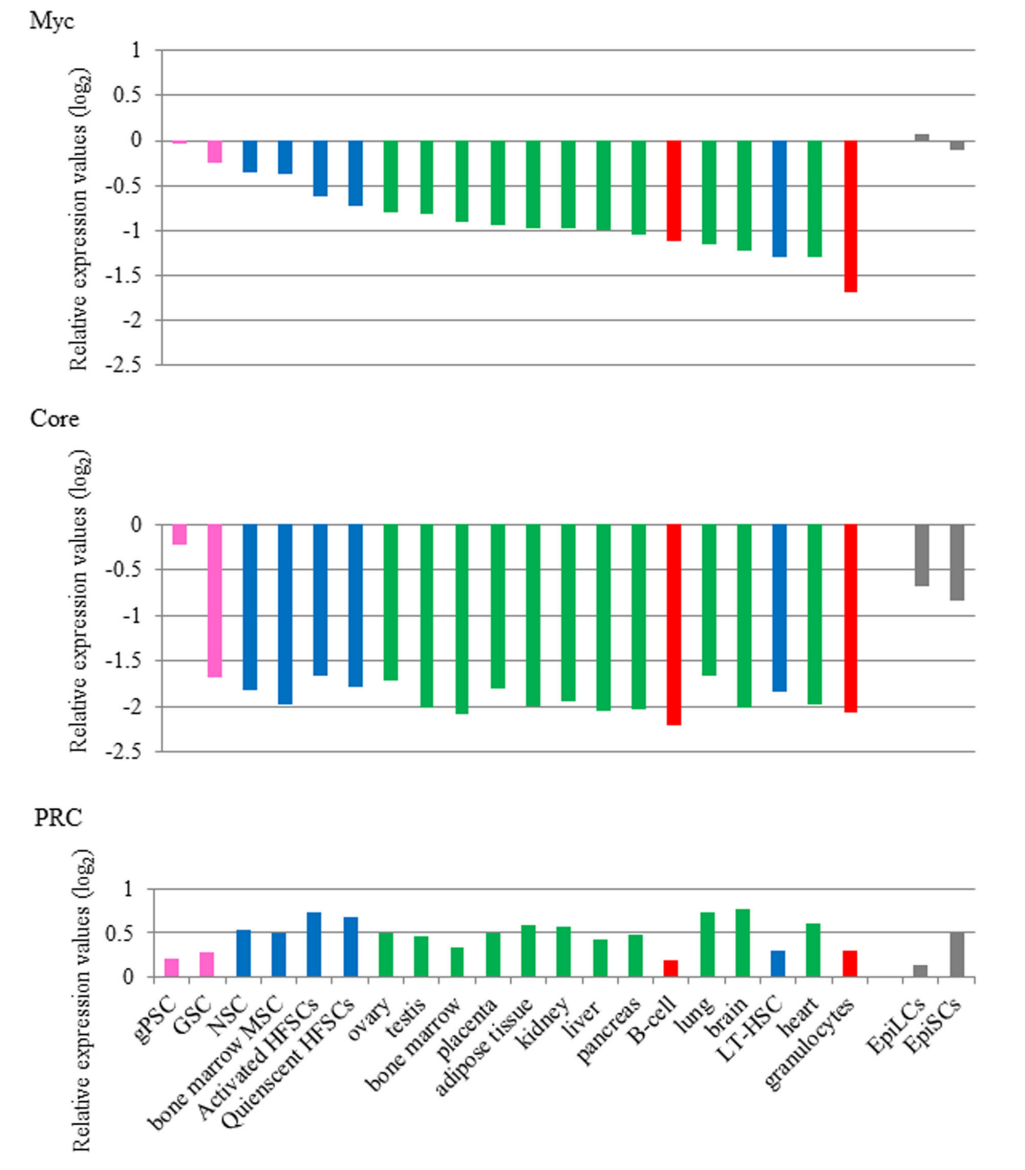
Highly specific activation of Core and Myc modules and repression of the PRC module in pluripotent cells. Publicly available DNA microarray data for 20 different tissue/somatic cell and stem cell types were obtained from the NCBI GEO database. To compare the same sets of genes used in Figures 1 and 2, data obtained using the same DNA microarray platform (Mouse Expression Array 430 platform, Affymetrix) by Hayashi et al. [29] were selected from the database. Average gene expression values (log_2_) of Core (upper panel), Myc (meddle panel), and PRC (lower panel) modules in each sample were calculated using those in ESCs as references. The data were aligned in an ordered fashion based on the value of average Myc module gene expression in which a sample showing the highest score, i.e., gPSC, was put at the left end of graph. The accession numbers of the obtained DNA microarray data are listed in the Materials and Methods. Data from germline stem cells and their derivatives, somatic stem cells, tissues, terminally differentiated hematopoietic cells and EpiSCs/EpiLCs are indicated by pink, blue, green, red, and gray bars, respectively, in the graph.

Because Myc module genes maintained high levels of expression not only in ESCs, but also in another pluripotent cell type, i.e., EpiSCs, we investigated whether a high level of Myc module gene expression is specific to pluripotent cells or widely observed in many cell types. To address this question, we obtained DNA microarray data for many cell types deposited in the NCBI GEO database. After normalization, we calculated the average expression levels of Core, Myc, and PRC module genes for 20 different cell types excluding ESCs, EpiSCs and EpiLCs (Figure 4). We found that all of the cell types except for gPSCs showed profoundly low expression of Core module genes compared with that in ESCs. Because gPSCs are generated by conferring pluripotency on GSCs, these results indicate that Core module gene expression is specific to pluripotent cells. For Myc module gene expression, none of the cell types except for gPSCs showed expression levels equivalent to those in ESCs. However, most stem cell types including GSCs, but excluding LT-HSCs appeared to show relatively higher levels of Myc module gene expression compared with those in other cell types. Thus, these results indicate that there is a good, if not perfect, correlation between the levels of Myc module gene expression and levels of undifferentiated state and/or proliferation rate of cells. We also found that all of the examined cell types showed relatively higher expression of PRC module genes compared with that in ESCs, although the expression levels were variable among the cell types.

### iPSCs and partial iPSCs show a highly similar expression profile of Myc module genes

**Figure 5:**
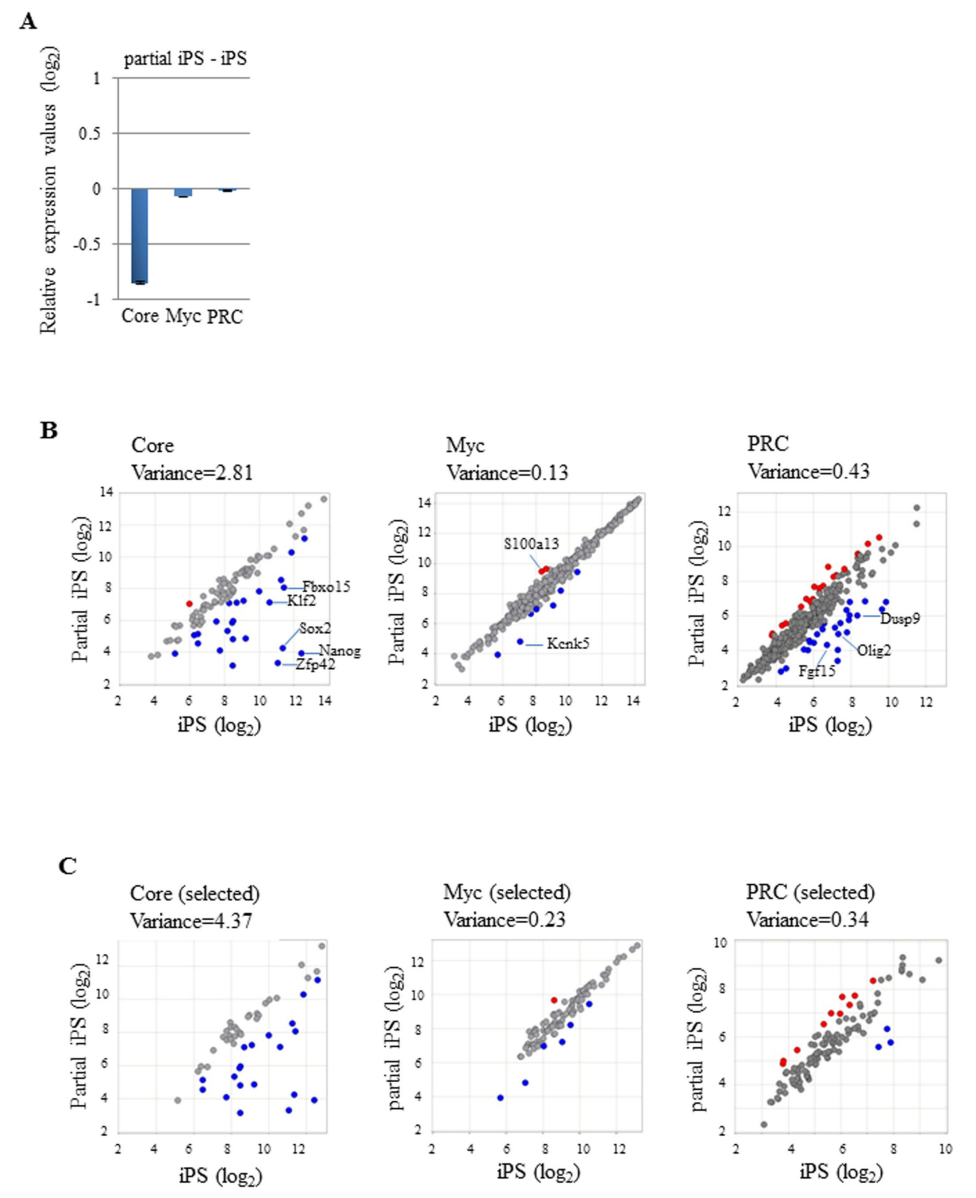
Examination of the expression of Core and Myc module genes in partial iPSCs. (A) Average gene expression values (log_2_) of Core, Myc, and PRC modules in partial iPSCs using those in iPSCs as references. The DNA microarray data deposited in GEO under GSE14012 had been obtained using the same platform (Mouse Expression Array 430 platform, Affymetrix) as that used by Hayashi et al. [29]. Therefore, the same sets of Core, Myc, and PRC module genes used in Figures 1, 2 and 4 could be used for the analyses. (B) Scatter plots of the expression of Core (left), Myc (middle), and PRC (right) module genes in partial and genuine iPSCs. The variance value was calculated and is shown for each scatter plot. Red and blue spots correspond to genes with more than 2-fold higher or lower expression in partial iPSCs than that in iPSCs, respectively. Gene symbols corresponding to red and blue are listed in Table S6. (C) Scatter plots of the expression of the selected Core (left), Myc (middle), and PRC (right) module genes in partial and genuine iPSCs. The same sets of genes used in Figure 1C were used for the analyses. The variance value was calculated and is shown for each scatter plot. Red and blue spots indicate as described in B.

The striking similarity in the gene expression of individual Myc module members among ESCs, EpiSC, and EpiLCs prompted us to investigate the expression profile of these genes in partial iPSCs that exhibit high expression of Myc module genes [20]. We used DNA microarray data of iPSCs and partial iPSC reported by Sridharan et al. [31] in GEO under GSE14012. Consistent with a previous report [20], Core module gene expression was very low in partial iPSCs, but Myc module gene expression levels were almost equivalent to those in iPSCs (Figure 5A). We also found that the average expression levels of PRC module genes were comparable in these cell types. Next, we compared individual gene expression levels of Core, Myc, and PRC module genes using scatter plots (Figure 5B). As expected, many Core module genes showed much lower expression in partial iPSCs compared with that in genuine iPSCs. Twenty-four out of 99 genes (24.2%) showed more than 2-fold lower expression levels in partial iPSCs, while only one Core module gene (1.0%) showed higher expression in partial iPSCs. A scatter plot comparing expression levels of Myc module genes demonstrated that there was at least equivalent or a higher level of similarity in the expression profiles of Myc module genes in partial and genuine iPSCs compared with that observed in the comparison between ESCs and EpiSCs/EpiLCs. We found that only two (0.5%) and seven (1.6%) genes in partial iPSCs showed higher or lower expression values compared with those in genuine iPSCs, respectively. Therefore, 97.9% of Myc module genes showed comparable expression in partial and genuine iPSCs. As shown in Figure 5C, essentially the same conclusion was attained from analyses of the selected genes used in Figure 1C, which showed more than 2-fold higher and lower expression of Core/Myc and PRC module genes, respectively, in iPSCs than that in MEFs. Similar to the analyses to compare gene expression of Core, Myc, and PRC module genes between ESCs and EpiSCs/EpiLCs, we examined the expression of the same set of housekeeping genes used in Figure S4A to validate our normalization of the deposited gene expression data. As a result, the housekeeping genes showed comparable levels of expression between partial and genuine iPSCs (Figure S4B).

### Direct evidence of the dependence of Myc expression for self-renewal of partial iPSCs

**Figure 6:**
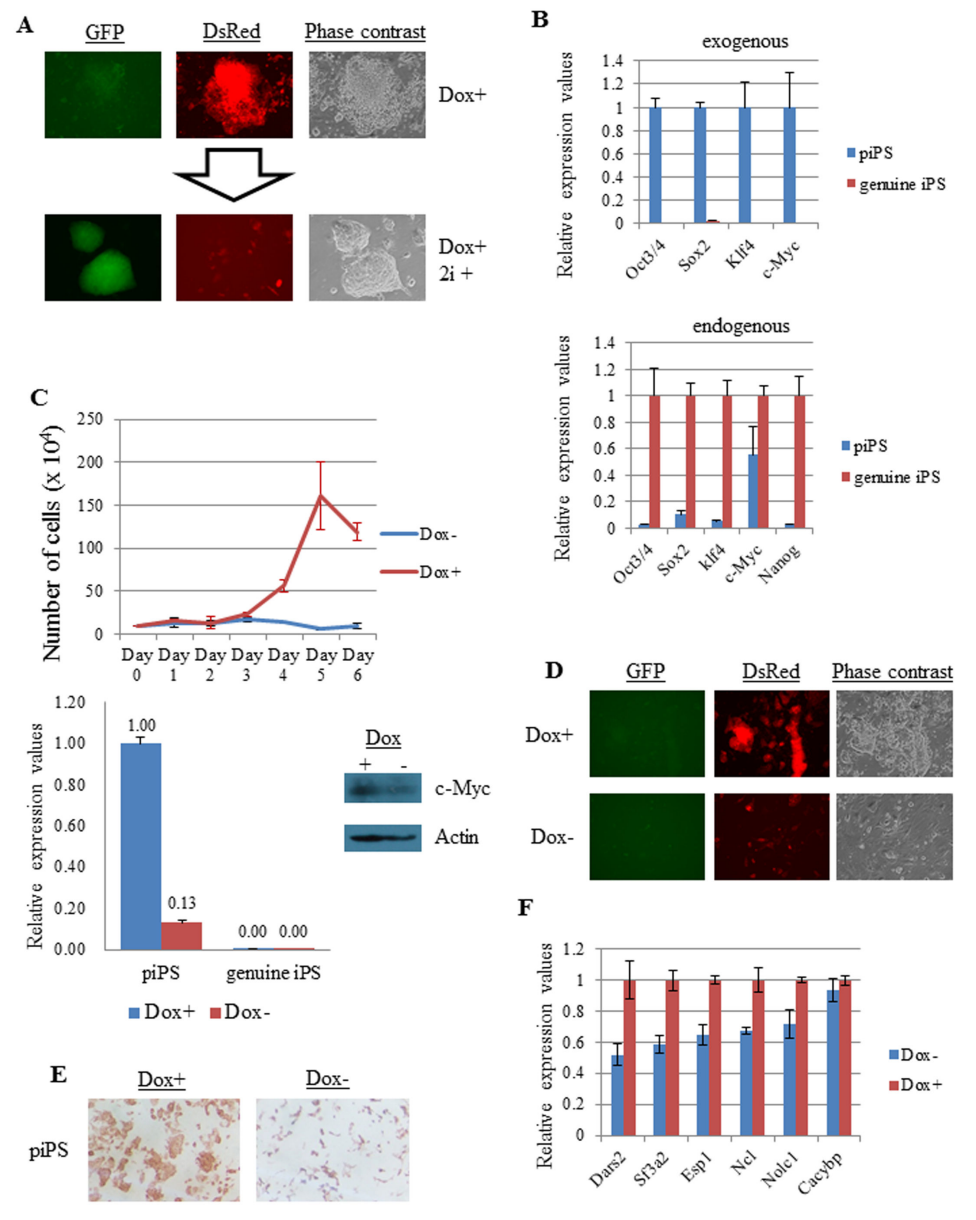
Dependence of c-Myc overexpression for robust proliferation of partial iPSCs. (A) Fluorescence and bright field microscopic images of partial iPSC clone 55 (upper panel) and the same cells subjected to 5 days of exposure to 2i (MAPK and GSK3β inhibitors) for conversion to genuine iPSCs. Red fluorescence corresponds to DsRed expression from a retrovirus also carrying the c-Myc gene under the control of a tTA-responsive element-containing promoter, whereas green fluorescence indicates expression of the *Nanog-GFP* reporter [26]. (B) Quantitative RT-PCR analyses of the expression of endogenous (end) and exogenous (exo) reprogramming factor genes, and the endogenous *Nanog* gene in partial iPSCs and those converted to genuine iPSCs by exposure to the 2i condition. Exogenous expression of reprogramming factors in partial iPSCs was arbitrarily set to one (upper panel), whereas endogenous expression of those in genuine iPSCs generated from partial iPSCs was set to one (lower panel). (C) Partial iPSCs were cultured in the presence or absence of Dox that allowed overexpression of c-Myc. Cell numbers were counted at the indicated days. The number of partial iPSCs at day 0 was arbitrarily set to one. Lower left panel shows the expression levels of the exogenous c-Myc gene in partial and genuine iPSCs that were cultured in the absence or presence of Dox. Although expression values of exogenous c-Myc of both Dox-treated and –untreated genuine iPSCs are indicated as 0.00, actual values of those are 1.3x10^-3^ and 1.4x10^-4^, respectively. Lower right panel shows the western blot analysis of total (exogenous and endogenous) c-Myc protein in Dox-treated and untreated partial iPSCs. (D) Bright field and fluorescence images of partial iPSC clone 55 that was cultured with or without Dox. (E) Alkaline phosphatase staining of Dox-treated and untreated partial iPSCs . (F) Myc module gene expression in Dox-treated and untreated partial iPSCs. Six genes (*Dars2*, *Sf3a2*, *Esp1*, *Nc1*, *Nolc1*, and *Cacybp*) were arbitrarily chosen from the selected Myc module gene set (98) showing more than 2-fold higher expression in iPSCs compared with that in MEFs (Table S3). Expression changes caused by Dox withdrawal from the culture medium was examined in partial iPSCs. The expression of each gene in Dox-treated partial iPSCs was arbitrarily set to one. *Dars2*, aspartyl-tRNA synthetase; *Sf3a2*, splicing factor 3a (subunit2); *Esp1*, exocrine gland-secreting peptide 1; *Nolc1*, nucleolar and coiled-body phosphoprotein 1; *Cacybp*, calcyclin-binding protein.

Strong conservation of the expression profile of Myc module genes among ESCs, EpiSCs/EpiLCs, and partial iPSCs suggests that these genes play specific biological roles that are commonly important among these cell types. To address this question directly, it would be important to observe phenotypic changes associated with down-regulation of expression of Myc module genes. One possible experiment to do is to see the consequence of deficiency of *Myc* gene expression which substantially contributes to sustaining expression of Myc module genes. However, the Myc family is comprised of three highly related proteins (c-Myc, N-Myc, and L-Myc) and all three Myc proteins are expressed in ESCs, EpiSCs, and EpiLCs. Therefore, to avoid the functional redundancy, expression of all three Myc members must be impaired in these cells, but such experiment is rather technically challenging. However, a high level of Myc expression in partial iPSCs is sustained by exogenous expression of the *c-Myc* gene and we hypothesized that this exogenous *c-Myc* gene substantially participates in sustaining high expression level of Myc module genes. Therefore, in order to control exogenous Myc expression level in partial iPSCs and examine its consequence on these cells, we generated partial iPSCs in which exogenous c-Myc expression was overexpressed under the control of the tet-on system. We generated these partial iPSCs by infection of MEFs with retroviruses carrying either Oct3/4, Sox2, Klf4, or rtTA genes together with a virus carrying both c-Myc and DsRed cDNAs. To distinguish between partial and genuine iPSCs, we used MEFs carrying the *GFP* gene under the control of the *Nanog* gene promoter, which would be highly expressed in iPSCs but not in partial iPSCs or MEFs [26]. Expression of *Oct3/4*, *Sox2*, *Klf4*, *DsRed* and *tTA* genes was driven by constitutively active promoters, while a rtTA responsive element-containing promoter mediated *c-Myc* gene expression. Therefore, a sufficiently high level of c-Myc expression was obtained only when Dox was added to the culture medium. A total of 100 independent cell colonies were obtained by iPSC induction of MEFs that were positive for DsRed but negative for the *Nanog-GFP* reporter. Among these colonies, one clone (#55) was selected based on several criteria including stable propagation and efficient conversion to GFP-positive genuine iPSCs by exposure to 2i plus LIF [28], but no tendency for spontaneous conversion to iPSCs in empirical mouse ESC medium without 2i. Figure 6A shows the 2i and LIF-mediated conversion of partial iPSCs to genuine iPSCs. We also confirmed the conversion by examining the expression of exogenous and endogenous pluripotency marker genes by quantitative RT-PCR (Figure 6B). As shown in Figure 6C, the partial iPSC clone was able to propagate robustly in the presence of Dox that allowed abundant *c-Myc* gene expression. However, this partial iPSC clone failed to proliferate in the absence of Dox. We also performed these experiments with genuine iPSCs generated from partial iPSC clone 55 and their parent MEFs (Figure S8A) and confirmed that neither genuine iPSCs nor MEFs showed Dox-dependent cell growth. Dox-independent cell proliferation of genuine iPSCs was expected because the retrovirus-based tet-on system was inactivated after transition to iPSCs (Figure 6B). Figure 6D shows the microscopic observation of Dox-treated and untreated partial iPSCs, and Figure 6E shows the positivity of partial iPSCs for alkaline phosphatase activity, an indicator of induction but not completion of the reprogramming process [47,48]. Genuine iPSCs generated by exposure of partial iPSCS to the 2i condition, but not MEFs, were also positive for alkaline phosphatase (Figure S8B). We also noted that untreated partial iPSCs showed some alkaline phosphatase activity (Figure 6E). We assumed that this observation was caused by endogenous c-Myc protein and/or “leaky” expression of exogenous c-Myc, which was evident even in the absence of Dox (Figure 6C). To examine whether exogenously expressed c-Myc protein in Dox-treated partial iPSCs was integrated into the endogenous Myc module network, we arbitrarily chose six genes among the selected Myc module genes (98) listed in Table S3 and examined their dependence on Dox for expression by quantitative RT-PCR. Based on these analyses, we found that five out of six genes showed Dox-dependent expression (Figure 6F). Taken together, these results strongly support our hypothesis that, similar to ESCs, a high level of Myc expression is crucial to sustain the self-renewal property of partial iPSCs through modulation of Myc module gene expression.

## Discussion

In this study, we examined the expression of individual Core, Myc, and PRC module genes in ESCs, EpiSCs, EpiLCs, and partial iPSCs. An expected finding was that many of the Core module genes are highly expressed in ESCs, but a substantial number of genes show lower expression in EpiSCs, EpiLCs, and partial iPSCs. Such gene expression results in lowing the average expression levels of Core module genes in EpiSCs, EpiLCs, and partial iPSCs compared with that in ESCs. Another expected finding was that the number of PRC module genes showing higher expression in EpiSCs and EpiLCs than that in ESCs was larger than the number of genes showing the opposing trend. We obtained this result possibly because EpiSCs represent more developmentally advanced cells than ESCs. Alternatively, this difference reflects the difference in culture condition used between ESCs and EpiSCs. We also found that expression of Myc module genes in EpiSCs is equally active in ESCs. This finding for Myc module genes is rather unexpected, firstly because the Myc module subnetwork has not been previously reported to be active in these cells. More specifically, a previous report has demonstrated that, unlike mouse ESCs, c-Myc expression functions negatively in preservation of the undifferentiated state of human ESCs [42].

Similar to mouse ESCs/EpiSCs, we found that the majority of Myc module members maintain constant levels of gene expression during the transition between the two different states (naïve and primed) of human iPSCs. Our data also show that Myc module genes are highly expressed in EpiLCs that recapitulate the expression profile of *in vivo* mouse epiblast cells much more faithfully than that of EpiSCs. This observation indicates that prominent expression of Myc module genes in mouse EpiSCs and human iPSCs is not the result of adaptation associated with establishment as stable cell lines, but is physiologically relevant. 

Our analyses of partial iPSCs, which are considered to stray from the normal reprogramming route and become immortalized [25], also provided some interesting observations. These cells are already known to possess highly active expression of Myc module genes [20]. However, our data demonstrate for the first time that at least an equal or higher level of similarity in the gene expression of individual Myc module members is evident between partial and genuine iPSCs compared with that between ESCs and EpiSCs. Taken together, these results demonstrate that almost the same subset of genes contribute to elevation of the average expression values of Myc modules in ESCs/iPSCs, EpiSCs, EpiLCs, and partial iPSCs. Our data suggest that the commonly activated subset of Myc module genes is responsible for specific biological roles that are commonly important for these cell types. The results shown in Figure 4 indicate that Myc module genes show high levels of expression specifically in pluripotent cells. The fact that these genes are generally not strongly expressed in non-pluripotent cells, except for partial iPSCs, further corroborates this assumption. We [30,49] and others [50-52] have already demonstrated that Myc expression, which plays a central role in supporting the expression of Myc module genes, is crucially important to sustain pluripotency and self-renewality of ESCs. Therefore, we addressed the requirement of Myc expression to sustain the partial iPSC state by generating partial iPSCs in which c-Myc is overexpressed in a Dox-dependent manner. As a result, our data indeed demonstrate the requirement of Myc overexpression to support the self-renewal property of partial iPSCs.

 In summary, we found that Myc module genes are highly expressed in EpiSCs and EpiLCs as well as in ESCs/iPSCs and partial iPSCs, and an extreme similarity is evident even when expression of individual Myc module genes is compared among these cell types. Furthermore, our data unequivocally demonstrate that a high level of Myc expression is at least important for sustaining the self-renewal properties of partial iPSCs as has been demonstrated for ESCs [30,50-52]. We hope that our data will serve as an important step toward complete elucidation of the physiological roles of Myc module genes, and the identification of genes among them that are crucial for such biological functions.

## Supporting Information

Figure S1 **Comparison of the expression of Core, Myc, and PRC module genes between iPSCs and MEFs.** (A) Average gene expression values (log_2_) of Core, Myc, and PRC module genes in iPSCs using those in MEFs as references. Data deposited by Sridharan et al. [31] were used for the analyses. (B) Scatter plot analyses of Core, Myc, and PRC module genes between iPSCs and MEFs. Red and blue spots indicate as described in Figure 1B. Genes marked with red among Core and Myc module genes and those marked with blue among PRC module genes are listed in Table S2 and were used for the analyses shown in Figures 1C, 2C, 3C, and 5C.
(TIF)

Figure S2 **GSEAs of Core (left), Myc (middle), and PRC (right) modules** Data from EpiSCs were shown using those from ESCs as references.
(TIF)

Figure S3 **Comparison of the expression of Core, Myc, and PRC module genes between ESCs and ESC-derived EpiSCs.** (A) Average gene expression values (log_2_) of Core, Myc, and PRC module genes in EpiSCs using those in ESCs as references. Data deposited by Rugg-Gunn et al. [32] (101 Core, 432 Myc, and 493 PRC module genes) were used for the analyses. The data lacked information for the expression levels of 10 Core, 71 Myc, and 67 PRC module genes. (B) Comparison of the expression of individual Core, Myc, and PRC module genes between ESCs and EpiSCs as described in Figure 1B. (C) Comparison of the expression of selected Core, Myc, and PRC module genes. Left, middle, and right scatter plots show the expression values of the selected Core, Myc, and PRC module genes listed in Table S2, respectively, in ESCs and EpiSCs. Red and blue spots indicate as described in Figure 1B. The variance value was calculated and is shown for each scatter plot.
(TIF)

Figure S4 **Comparison of housekeeping gene expression.** (A) Eight different housekeeping genes were arbitrarily selected to compare their expression values in EpiSCs and EpiLCs using those in ESCs as references in the downloaded data for the analyses shown in Figures 1 and 2 (GSE30056). Actb, beta-actin; Atp5f1, ATP synthase, H+ transporting, mitochondrial FoFo complex, subunit B1; Gapdh, glyceraldehyde-3-phosphate dehydrogenase; Pgk1, phosphoglycerate kinase 1; Ppia, peptidylprolyl isomerase A; Rplp1, ribosomal protein, large, P1; Tbp, TATA binding protein; Ywhaz, tyrosine 3-monooxygenase/tryptophan 5-monooxygenase activation protein, zeta polypeptide. (B) The same set of housekeeping genes in (A) was used to examine their relative expression values in partial iPSCs compared with those in genuine iPSCs using the data deposited at NCBI GEO under the accession number GSE14012 that were used for the analyses shown in Figure 5.
(TIF)

Figure S5 **Comparison of expression levels of X-chromosome genes between ESCs and EpiSCs/EpiLCs.** Seven X-chromosome genes (*Abcd1, Atrx, Irak1, Kdm6a, Mecp2, Rlim, and Usp27x*) showing rather ubiquitous expression profile were randomly selected and compared the expression levels between ESCs and EpiSCs or EpiLCs using data set deposited under GSE30056.
(TIF)

Figure S6 **Quantitative RT-PCR for validation of DNA microarray data from differentially expressed Myc module genes in ESCs and EpiLCs.** (A) Comparison of the expression of three different Myc module genes (*Cdca71*, *Ldha*, and *Rab25*) by quantitative RT-PCR, which are highly expressed in EpiLCs compared with that in ESCs based on publicly available DNA microarray data (GSE30056). Expression of each gene in ESCs was arbitrarily set to one. (B) Comparison of the expression of three different Myc module genes (*D230025D16Rik*, *Fn1*, and *S100a13*) by quantitative RT-PCR, which are down-regulated during the transition from ESCs to EpiLCs based on the above DNA microarray data. Expression of each gene in ESCs was arbitrarily set to one. (C) Comparison of the expression of eight different Myc module genes (*Dars2, Nolc1, Mcm6, Sf3a2, Cacybp, Incenp, Espl1, and Ncl*) by quantitative RT-PCR, which are supposed to be equivalent in their expression levels between ESCs and EpiLCs based on the above DNA microarray data. Expression of each gene in ESCs was arbitrarily set to one.
(TIF)

Figure S7 **Comparison of expression levels of genes which would participate in controlling expression of Myc module genes between ESCs and EpiSCs/EpiLCs.** Expression data were extracted from data set deposited under GSE30056. In addition to expression data of seven genes (*Dmap1, E2F1, E2F4, Zfx, Max, c-Myc, N-Myc*) which are designated to be involved in controlling Myc module genes, expression data of L-Myc gene was also included.
(TIF)

Figure S8 **Analyses of expression levels of Core, Myc, and PRC module genes in exogenous reprogramming factor-independent naïve pluripotent human ESCs compared to those in primed state.** (A) Average gene expression values (log_2_) of Core, Myc, and PRC module genes in primed human iPSCs using those in human iPSCs converted to a naïve state as described by Gafni et al. [46]. Data from 71 Core, 327 Myc, and 422 PRC module genes deposited in GEO under GSE46872 were used for the analyses. Data from 4 Core, 28 Myc, and 29 PRC module genes are not available in the deposited data sets. (B) Comparison of the expression of individual Core, Myc, and PRC module genes between reprogramming factor-independent naïve and primed human ESCs. Left, middle, and right scatter plots show the expression values of individual Core, Myc, and PRC module genes, respectively, in naïve and primed human ESCs. Red and blue spots indicate genes with expression levels that are higher or lower by more than 2-fold in primed human iPSCs compared with those in naïve human iPSCs, respectively. The variance value was calculated and is shown for each scatter plot.
(TIF)

Figure S9 **Dox-independent cell growth of genuine iPSCs and their parental MEFs.** (A) Unlike Dox-dependent partial iPSC clone (#55), genuine iPSCs derived from partial iPSCs and their parental MEFs did not show Dox dependency for their growth. Cell numbers were counted as described in Figure 6C. Relatively slower cell proliferation of Dox-treated MEFs compared with that of untreated cells may represent non-specific toxicity of Dox in some cultured cells. Right panels show bright field and fluorescence images of iPSCs and MEFs cultured with or without Dox. GFP fluorescence in iPSCs indicates *Nanog-GFP* reporter expression that recapitulates endogenous *Nanog* gene expression, while no DsRed fluorescence irrespective of the presence or absence of Dox indicates silencing of retrovirus-mediated gene expression, which is one of the indicators of transition from partial to genuine iPSCs. (B) Alkaline phosphatase staining analyses of Dox-treated and untreated iPSCs and MEFs.
(TIF)

Table S1 **Sequence of primers used for RT-PCR analyses.** exo and end indicate exogenous and endogenous, respectively.
(TIF)

Table S2 **Lists of Core, Myc and PRC module genes which show differential expression levels between ESCs and EpiSCs.** Red letter indicates the genes which also show differential expression between ESCs and EpiLCs.
(TIF)

Table S3 **Lists of genes showing more than 2-fold higher (Core and Myc module genes) and lower (PRC module genes) expression in ESCs compared to MEFs.**
(TIF)

Table S4 **Lists of Core, Myc and PRC module genes which show differential expression levels between ESCs and EpiSCs.** Genes also listed in Table S2 are marked with red letter.
(TIF)

Table S5 **Lists of Core, Myc and PRC module genes which show differential expression levels between human iPSCs in primed state and those in naïve state.**
(TIF)

Table S6 **Lists of Core, Myc and PRC module genes which show differential expression levels between partial and genuine iPSCs.**
(TIF)
