# Supplementary figures and images for: Striking Similarity in the Gene Expression Levels of Individual Myc Module Members among ESCs, EpiSCs, and Partial iPSCs
Source: PLoS One. 2013 Dec 26;8(12):e83769. doi: 10.1371/journal.pone.0083769 (PMC3873369; doi:10.1371/journal.pone.0083769)

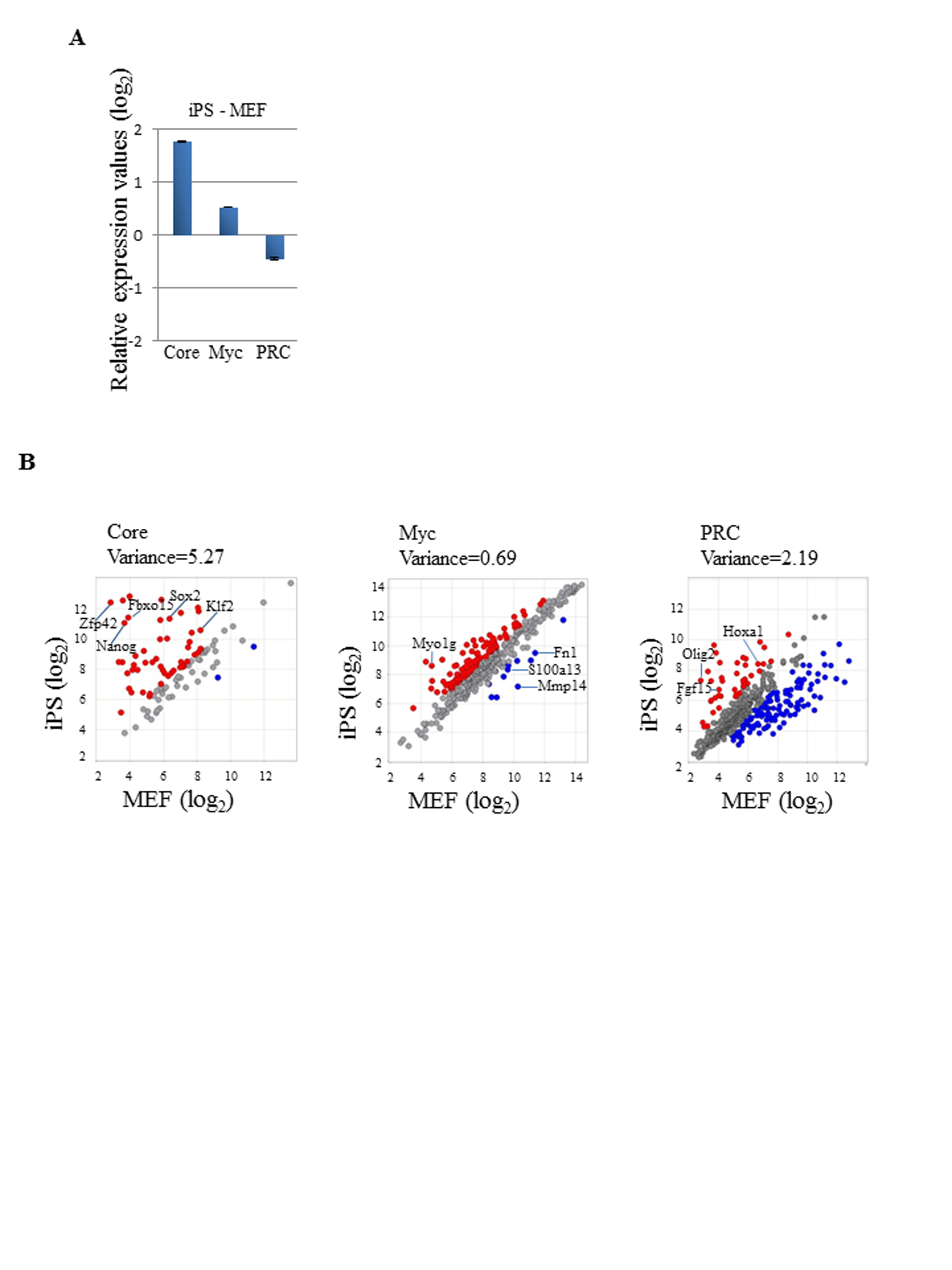

Supplement: Figure S1 — Comparison of the expression of Core, Myc, and PRC module genes between iPSCs and MEFs. (A) Average gene expression values (log2) of Core, Myc, and PRC module genes in iPSCs using those in MEFs as references. Data deposited by Sridharan et al. [31] were used for the analyses. (B) Scatter plot analyses of Core, Myc, and PRC module genes between iPSCs and MEFs. Red and blue spots indicate as described in Figure 1B. Genes marked with red among Core and Myc module genes and those marked with blue among PRC module genes are listed in Table S2 and were used for the analyses shown in Figures 1C, 2C, 3C, and 5C. (TIF) [file pone.0083769.s001.tif]

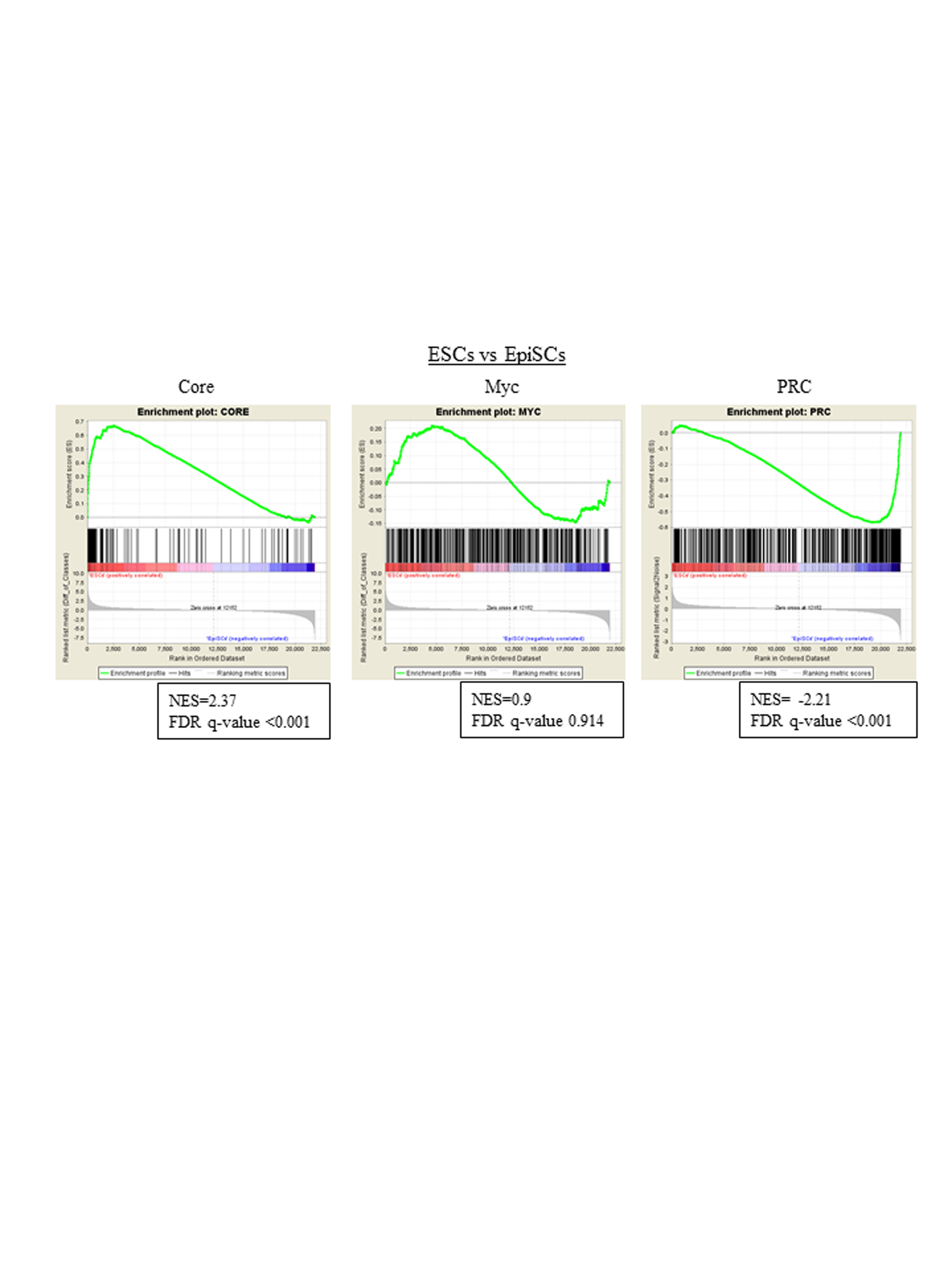

Supplement: Figure S2 — GSEAs of Core (left), Myc (middle), and PRC (right) modules Data from EpiSCs were shown using those from ESCs as references. (TIF) [file pone.0083769.s002.tif]

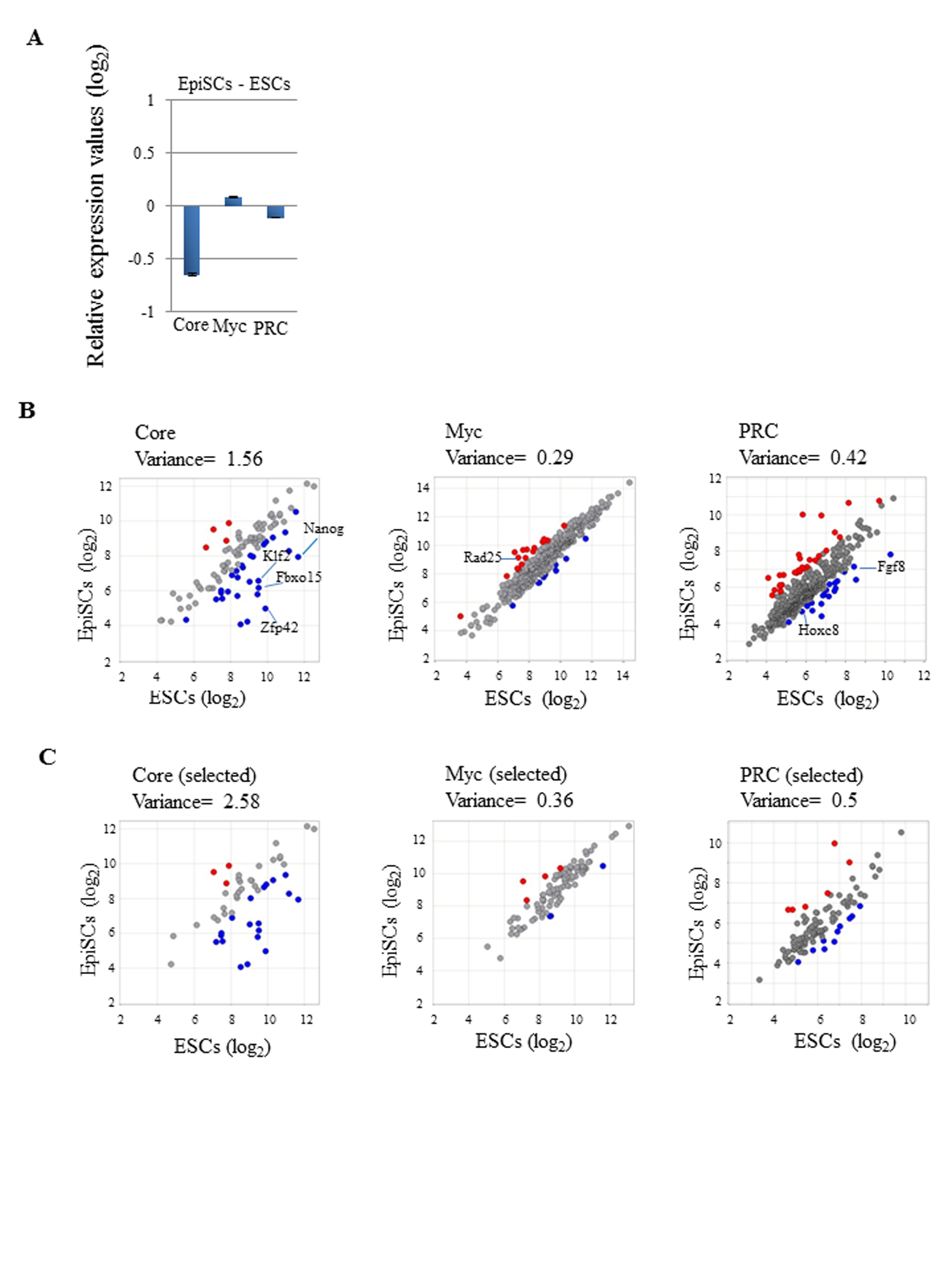

Supplement: Figure S3 — Comparison of the expression of Core, Myc, and PRC module genes between ESCs and ESC-derived EpiSCs. (A) Average gene expression values (log2) of Core, Myc, and PRC module genes in EpiSCs using those in ESCs as references. Data deposited by Rugg-Gunn et al. [32] (101 Core, 432 Myc, and 493 PRC module genes) were used for the analyses. The data lacked information for the expression levels of 10 Core, 71 Myc, and 67 PRC module genes. (B) Comparison of the expression of individual Core, Myc, and PRC module genes between ESCs and EpiSCs as described in Figure 1B. (C) Comparison of the expression of selected Core, Myc, and PRC module genes. Left, middle, and right scatter plots show the expression values of the selected Core, Myc, and PRC module genes listed in Table S2, respectively, in ESCs and EpiSCs. Red and blue spots indicate as described in Figure 1B. The variance value was calculated and is shown for each scatter plot. (TIF) [file pone.0083769.s003.tif]

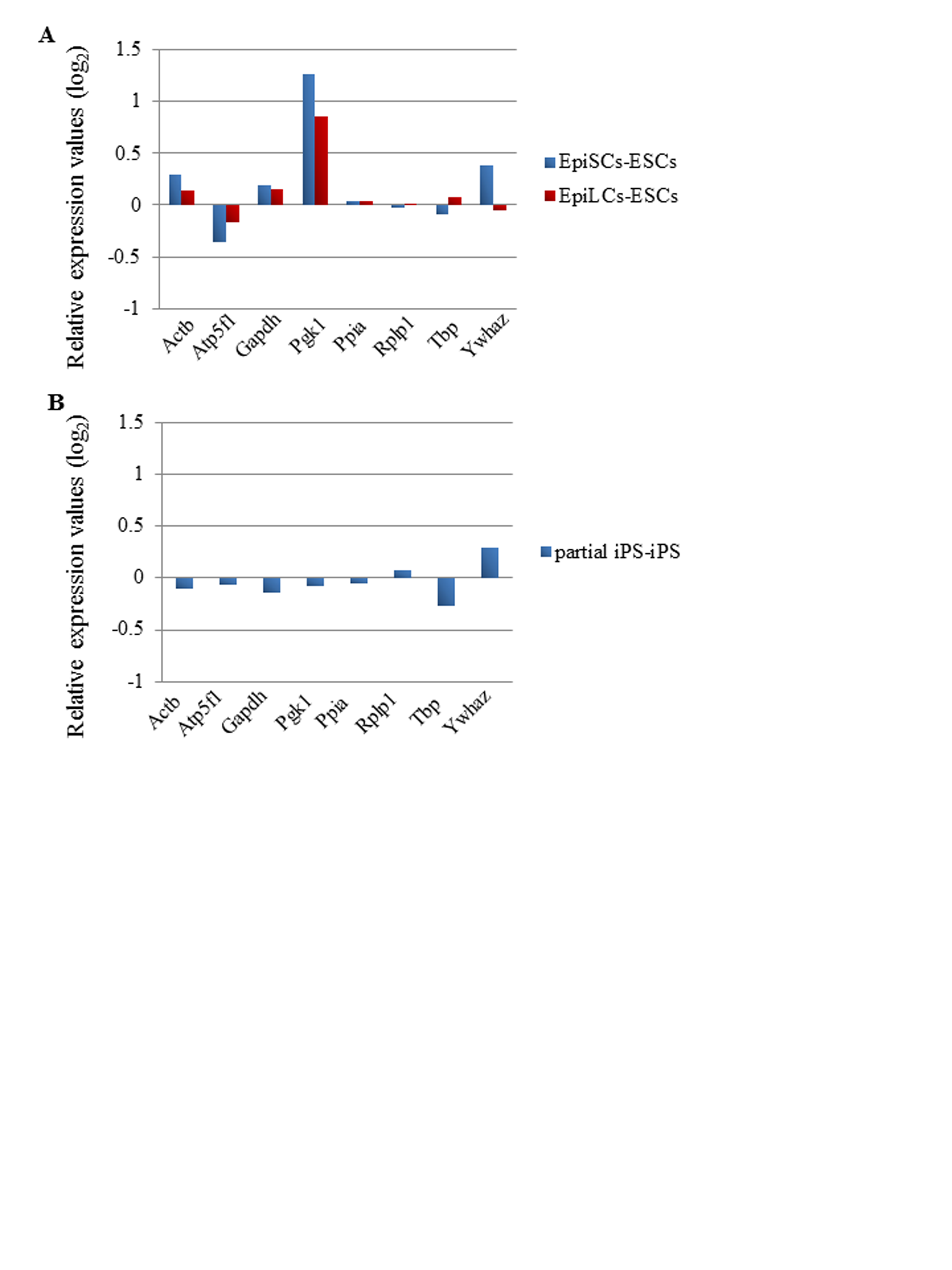

Supplement: Figure S4 — Comparison of housekeeping gene expression. (A) Eight different housekeeping genes were arbitrarily selected to compare their expression values in EpiSCs and EpiLCs using those in ESCs as references in the downloaded data for the analyses shown in Figures 1 and 2 (GSE30056). Actb, beta-actin; Atp5f1, ATP synthase, H+ transporting, mitochondrial FoFo complex, subunit B1; Gapdh, glyceraldehyde-3-phosphate dehydrogenase; Pgk1, phosphoglycerate kinase 1; Ppia, peptidylprolyl isomerase A; Rplp1, ribosomal protein, large, P1; Tbp, TATA binding protein; Ywhaz, tyrosine 3-monooxygenase/tryptophan 5-monooxygenase activation protein, zeta polypeptide. (B) The same set of housekeeping genes in (A) was used to examine their relative expression values in partial iPSCs compared with those in genuine iPSCs using the data deposited at NCBI GEO under the accession number GSE14012 that were used for the analyses shown in Figure 5. (TIF) [file pone.0083769.s004.tif]

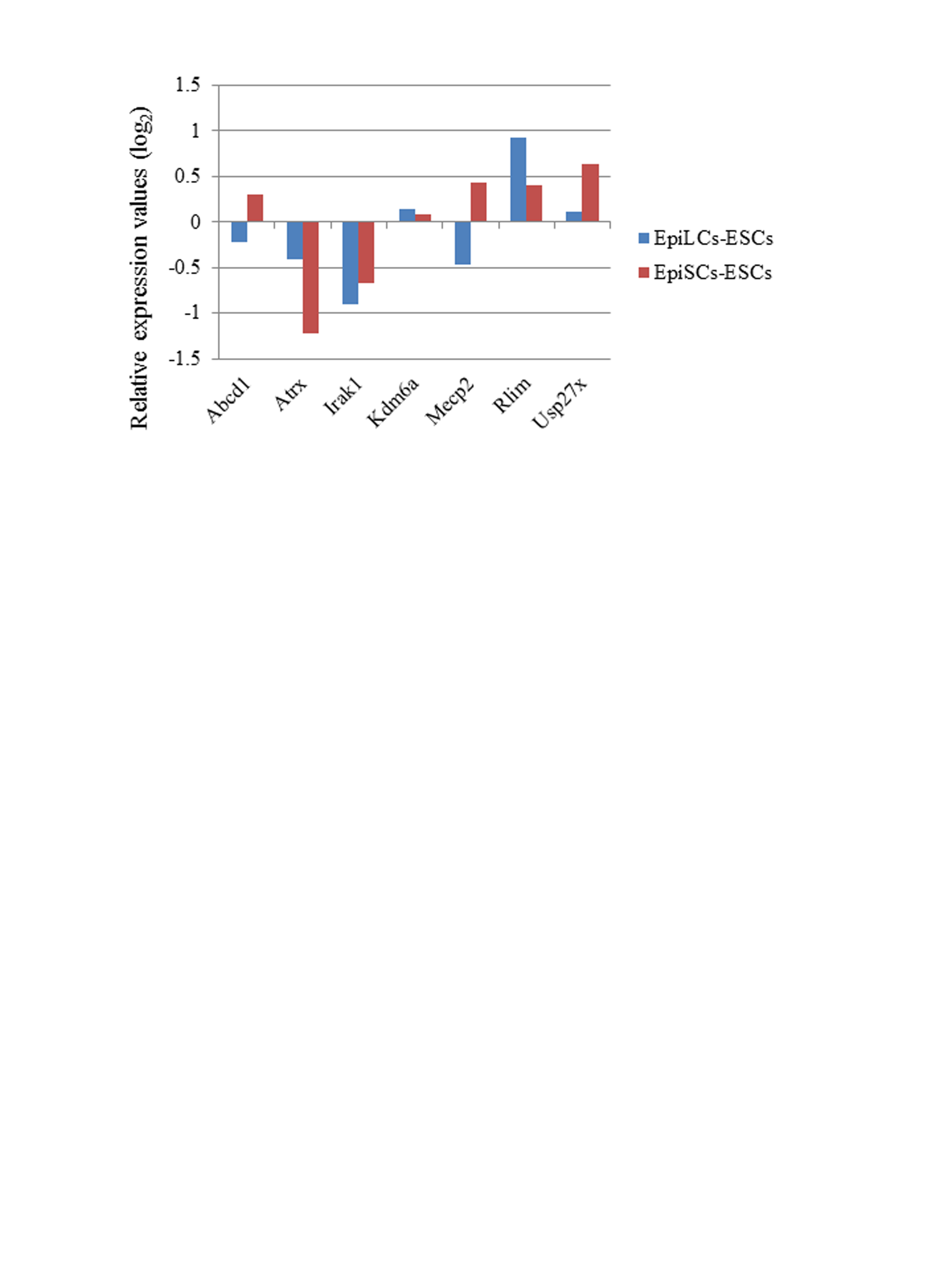

Supplement: Figure S5 — Comparison of expression levels of X-chromosome genes between ESCs and EpiSCs/EpiLCs. Seven X-chromosome genes (Abcd1, Atrx, Irak1, Kdm6a, Mecp2, Rlim, and Usp27x) showing rather ubiquitous expression profile were randomly selected and compared the expression levels between ESCs and EpiSCs or EpiLCs using data set deposited under GSE30056. (TIF) [file pone.0083769.s005.tif]

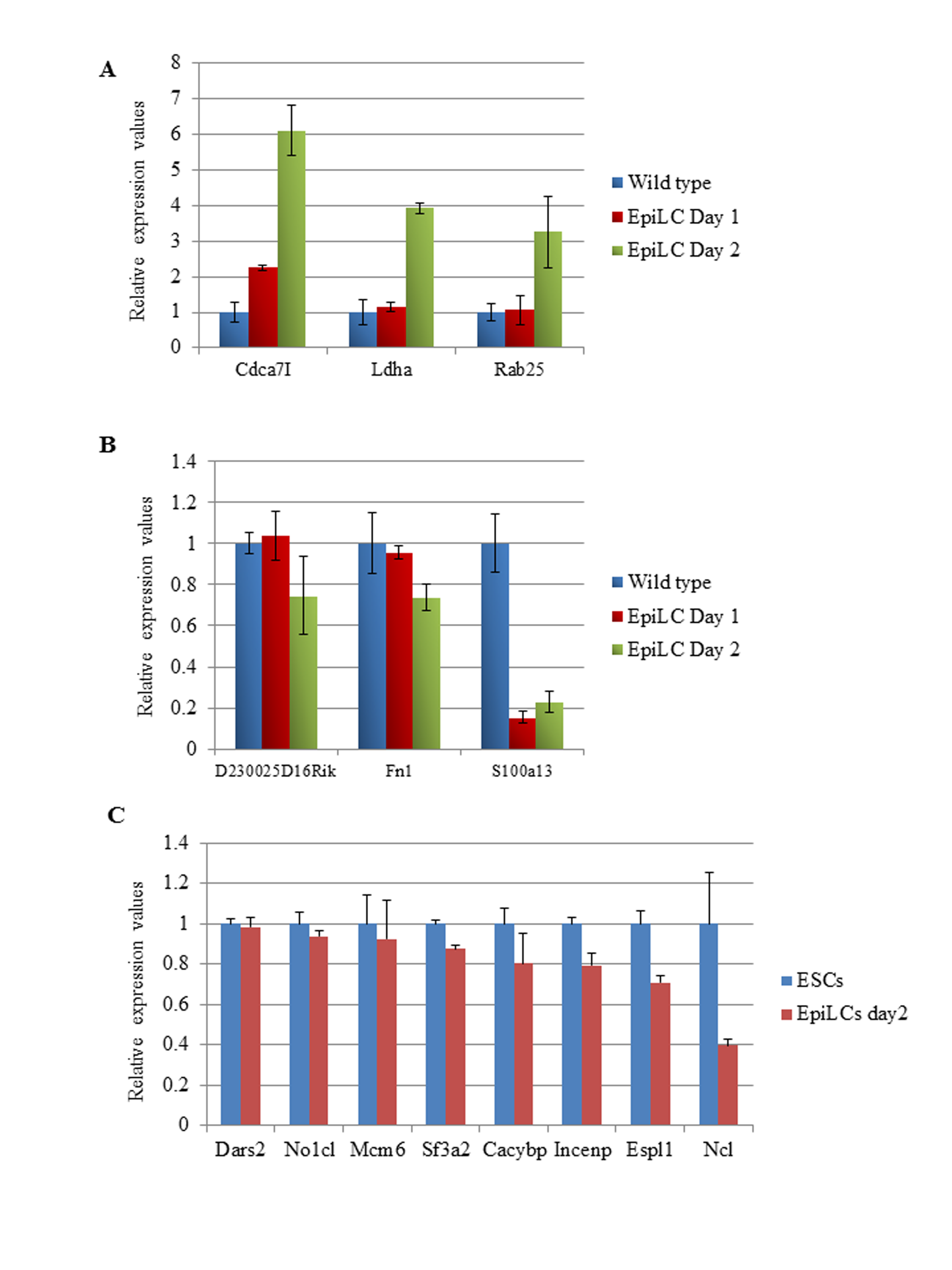

Supplement: Figure S6 — Quantitative RT-PCR for validation of DNA microarray data from differentially expressed Myc module genes in ESCs and EpiLCs. (A) Comparison of the expression of three different Myc module genes (Cdca71, Ldha, and Rab25) by quantitative RT-PCR, which are highly expressed in EpiLCs compared with that in ESCs based on publicly available DNA microarray data (GSE30056). Expression of each gene in ESCs was arbitrarily set to one. (B) Comparison of the expression of three different Myc module genes (D230025D16Rik, Fn1, and S100a13) by quantitative RT-PCR, which are down-regulated during the transition from ESCs to EpiLCs based on the above DNA microarray data. Expression of each gene in ESCs was arbitrarily set to one. (C) Comparison of the expression of eight different Myc module genes (Dars2, Nolc1, Mcm6, Sf3a2, Cacybp, Incenp, Espl1, and Ncl) by quantitative RT-PCR, which are supposed to be equivalent in their expression levels between ESCs and EpiLCs based on the above DNA microarray data. Expression of each gene in ESCs was arbitrarily set to one. (TIF) [file pone.0083769.s006.tif]

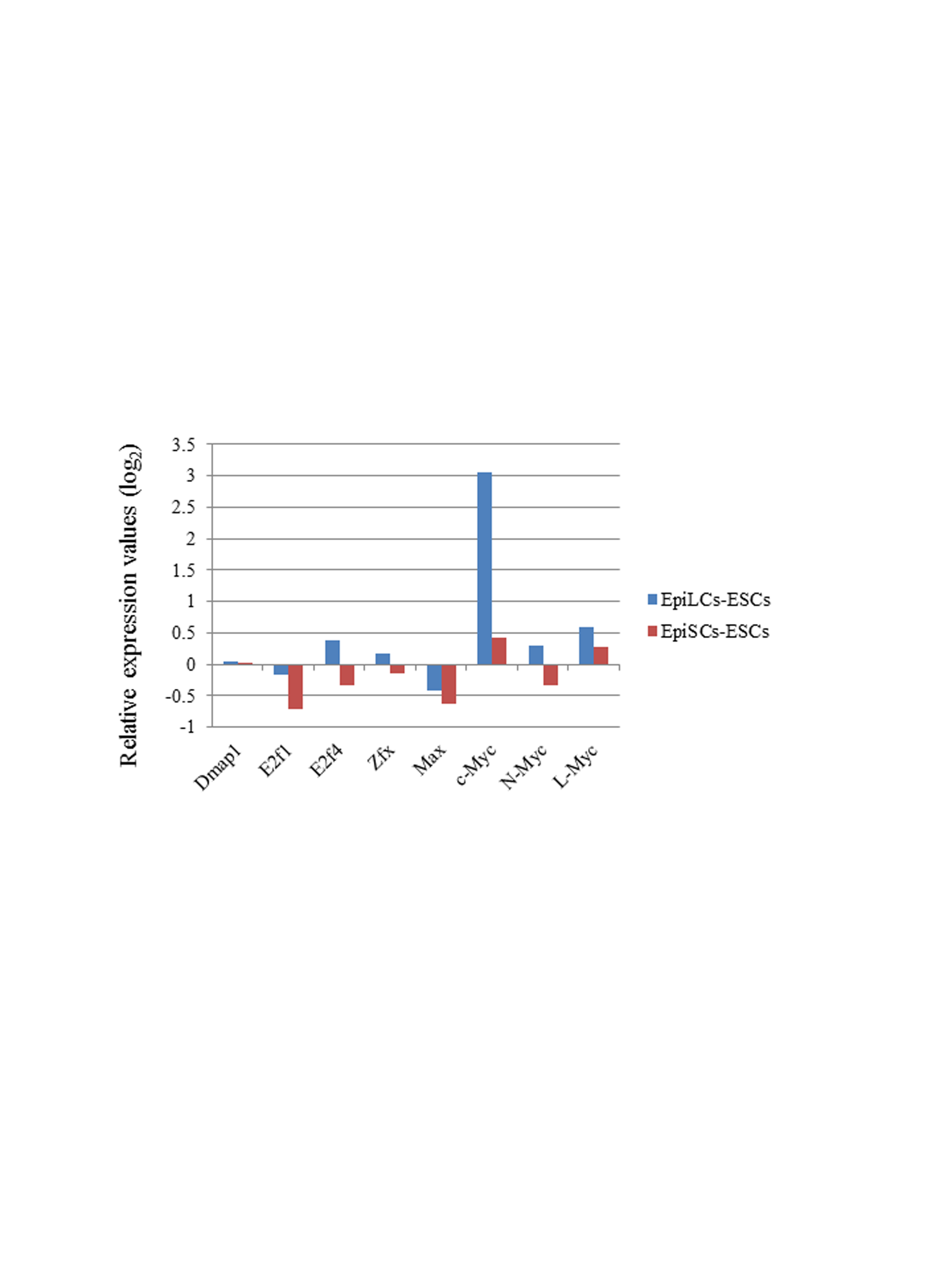

Supplement: Figure S7 — Comparison of expression levels of genes which would participate in controlling expression of Myc module genes between ESCs and EpiSCs/EpiLCs. Expression data were extracted from data set deposited under GSE30056. In addition to expression data of seven genes (Dmap1, E2F1, E2F4, Zfx, Max, c-Myc, N-Myc) which are designated to be involved in controlling Myc module genes, expression data of L-Myc gene was also included. (TIF) [file pone.0083769.s007.tif]

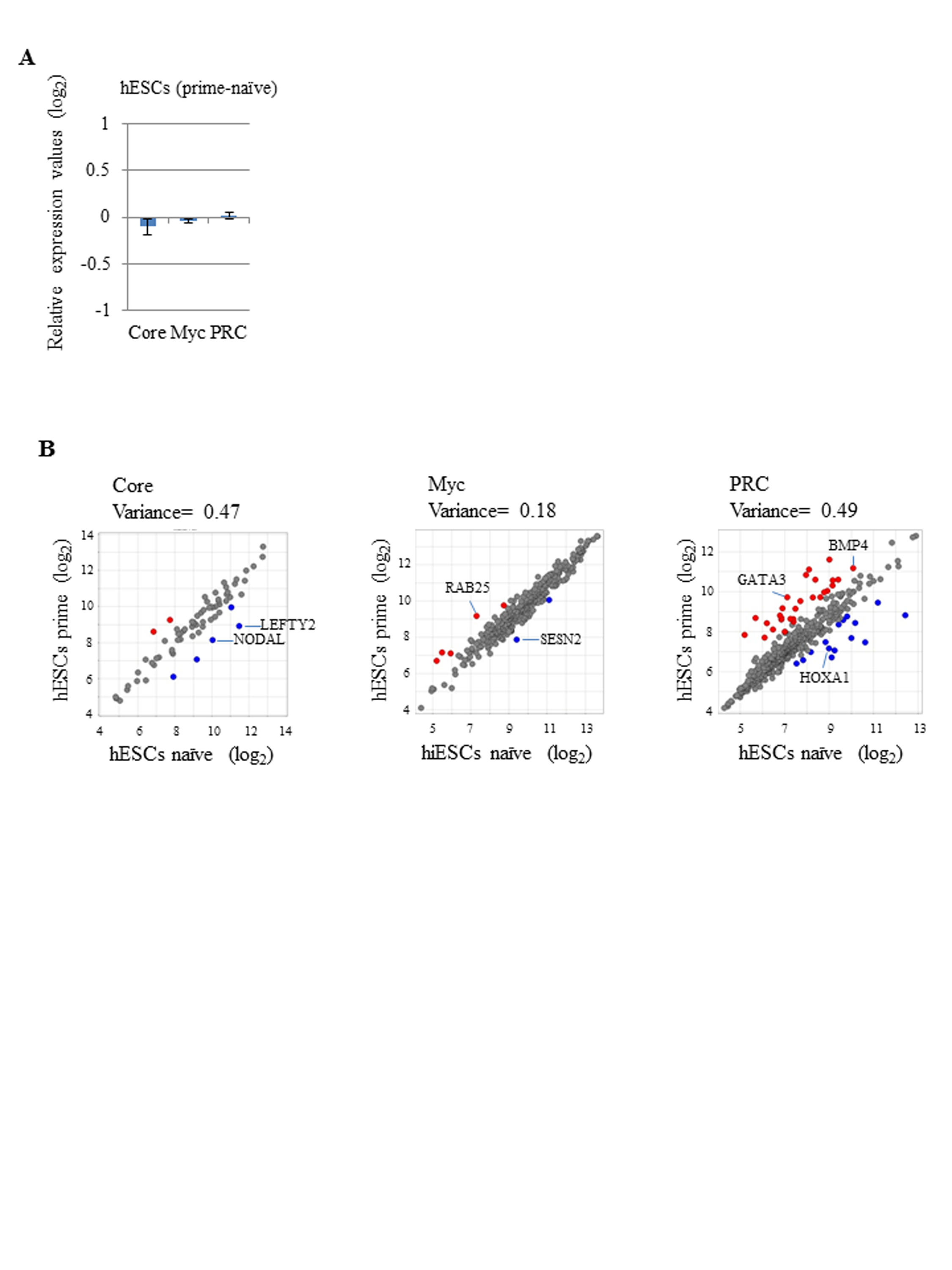

Supplement: Figure S8 — Analyses of expression levels of Core, Myc, and PRC module genes in exogenous reprogramming factor-independent naïve pluripotent human ESCs compared to those in primed state. (A) Average gene expression values (log2) of Core, Myc, and PRC module genes in primed human iPSCs using those in human iPSCs converted to a naïve state as described by Gafni et al. [46]. Data from 71 Core, 327 Myc, and 422 PRC module genes deposited in GEO under GSE46872 were used for the analyses. Data from 4 Core, 28 Myc, and 29 PRC module genes are not available in the deposited data sets. (B) Comparison of the expression of individual Core, Myc, and PRC module genes between reprogramming factor-independent naïve and primed human ESCs. Left, middle, and right scatter plots show the expression values of individual Core, Myc, and PRC module genes, respectively, in naïve and primed human ESCs. Red and blue spots indicate genes with expression levels that are higher or lower by more than 2-fold in primed human iPSCs compared with those in naïve human iPSCs, respectively. The variance value was calculated and is shown for each scatter plot. (TIF) [file pone.0083769.s008.tif]

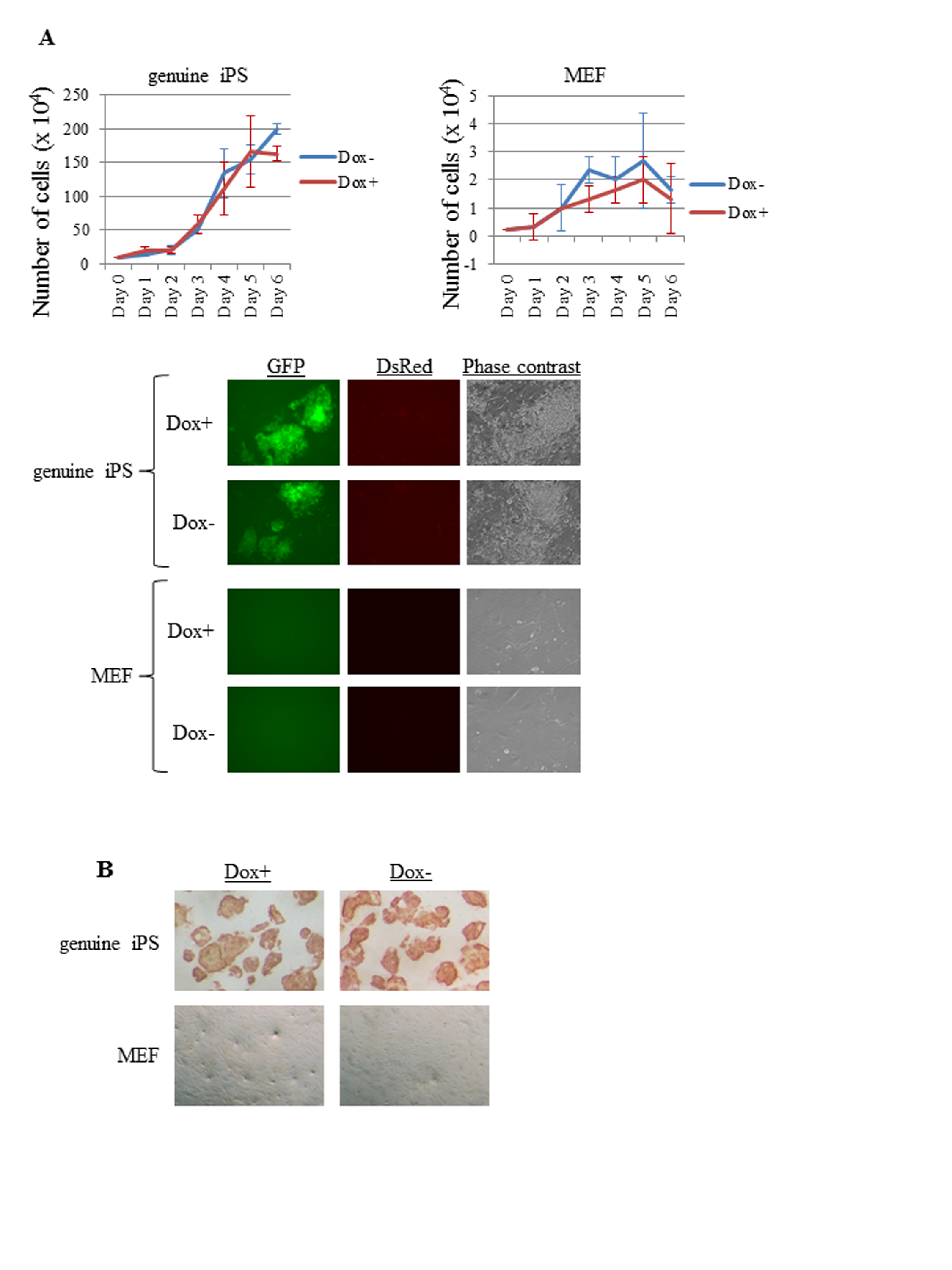

Supplement: Figure S9 — Dox-independent cell growth of genuine iPSCs and their parental MEFs. (A) Unlike Dox-dependent partial iPSC clone (#55), genuine iPSCs derived from partial iPSCs and their parental MEFs did not show Dox dependency for their growth. Cell numbers were counted as described in Figure 6C. Relatively slower cell proliferation of Dox-treated MEFs compared with that of untreated cells may represent non-specific toxicity of Dox in some cultured cells. Right panels show bright field and fluorescence images of iPSCs and MEFs cultured with or without Dox. GFP fluorescence in iPSCs indicates Nanog-GFP reporter expression that recapitulates endogenous Nanog gene expression, while no DsRed fluorescence irrespective of the presence or absence of Dox indicates silencing of retrovirus-mediated gene expression, which is one of the indicators of transition from partial to genuine iPSCs. (B) Alkaline phosphatase staining analyses of Dox-treated and untreated iPSCs and MEFs. (TIF) [file pone.0083769.s009.tif]

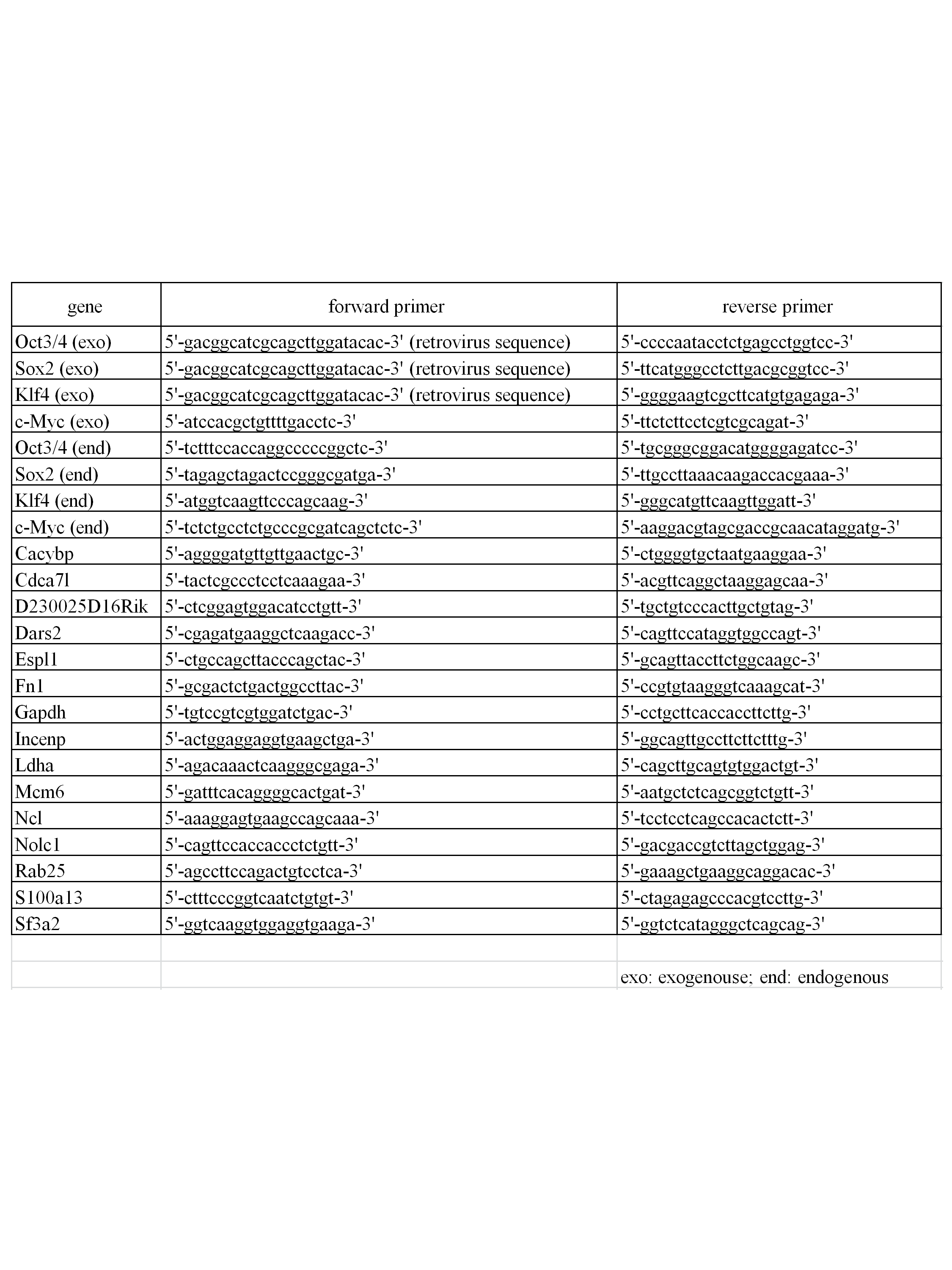

Supplement: Table S1 — Sequence of primers used for RT-PCR analyses. exo and end indicate exogenous and endogenous, respectively. (TIF) [file pone.0083769.s010.tif]

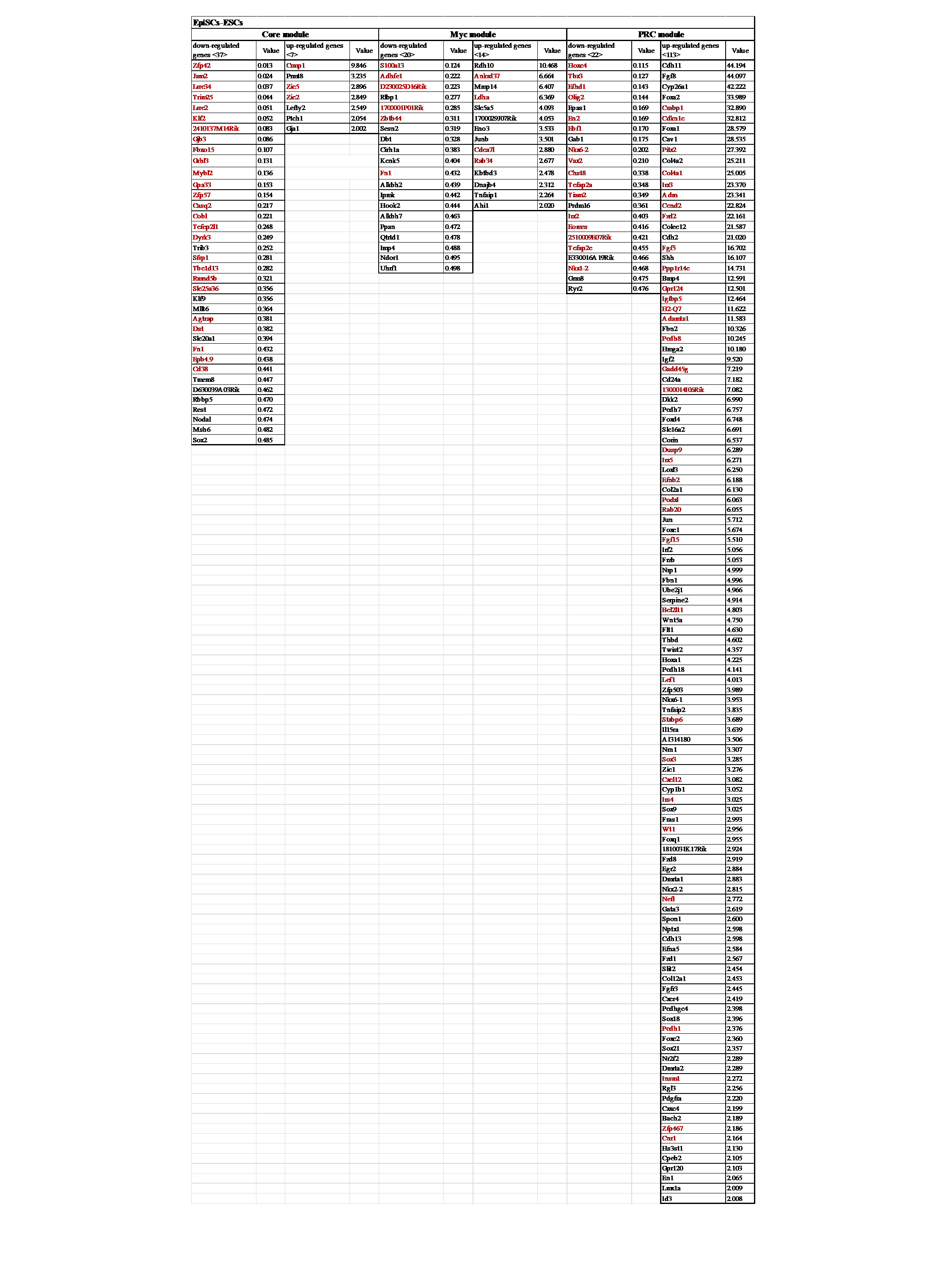

Supplement: Table S2 — Lists of Core, Myc and PRC module genes which show differential expression levels between ESCs and EpiSCs. Red letter indicates the genes which also show differential expression between ESCs and EpiLCs. (TIF) [file pone.0083769.s011.tif]

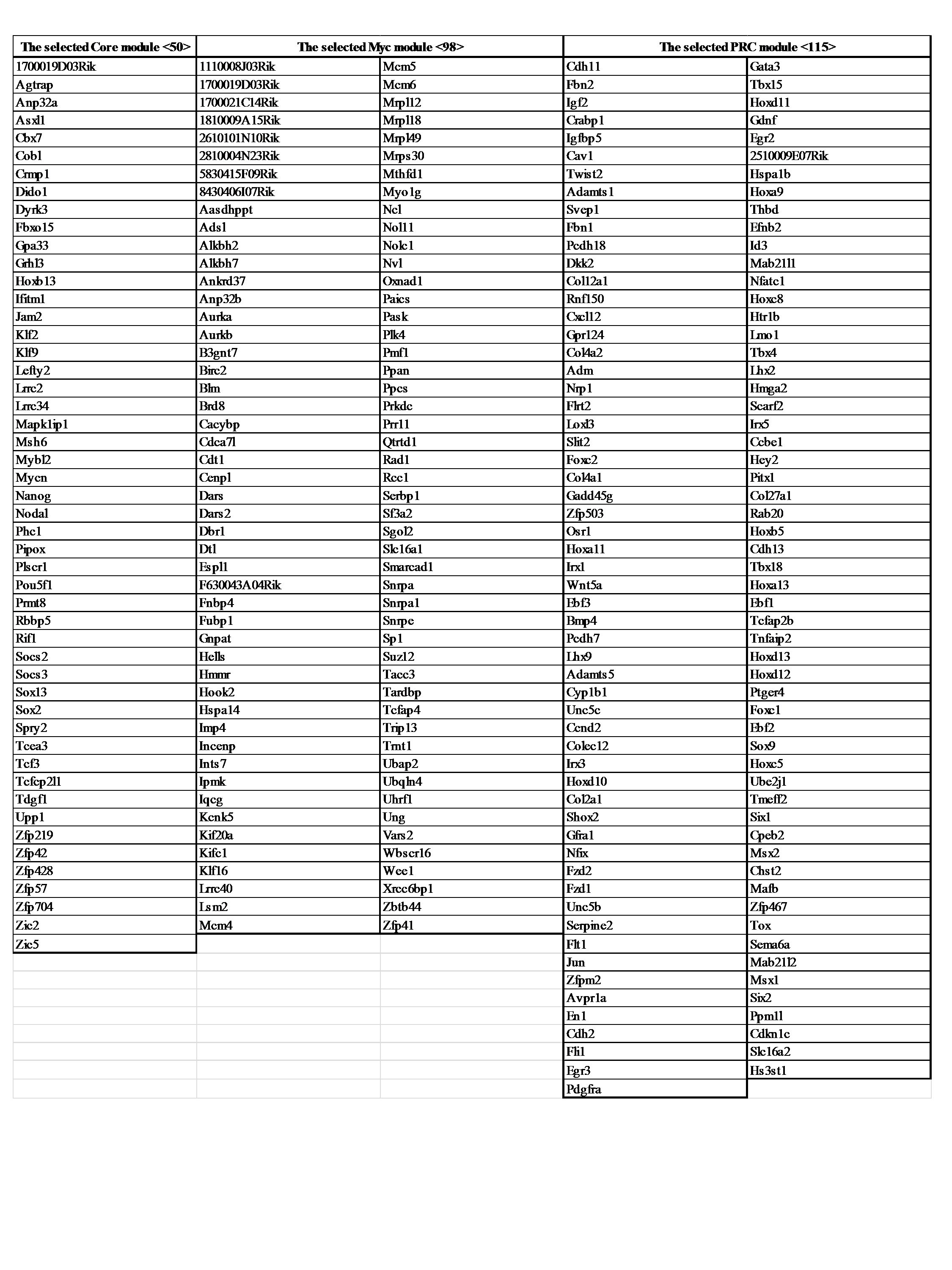

Supplement: Table S3 — Lists of genes showing more than 2-fold higher (Core and Myc module genes) and lower (PRC module genes) expression in ESCs compared to MEFs. (TIF) [file pone.0083769.s012.tif]

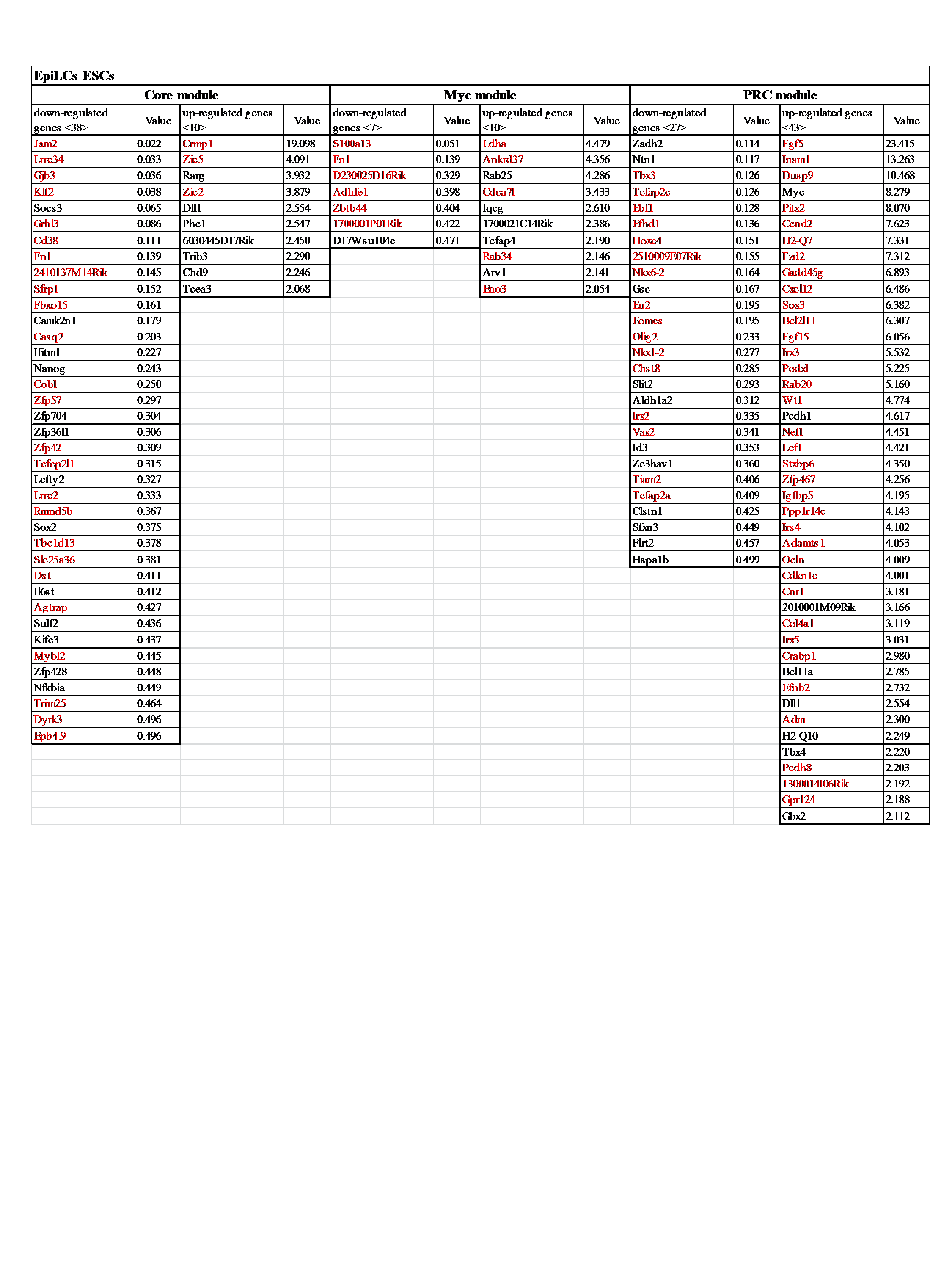

Supplement: Table S4 — Lists of Core, Myc and PRC module genes which show differential expression levels between ESCs and EpiSCs. Genes also listed in Table S2 are marked with red letter. (TIF) [file pone.0083769.s013.tif]

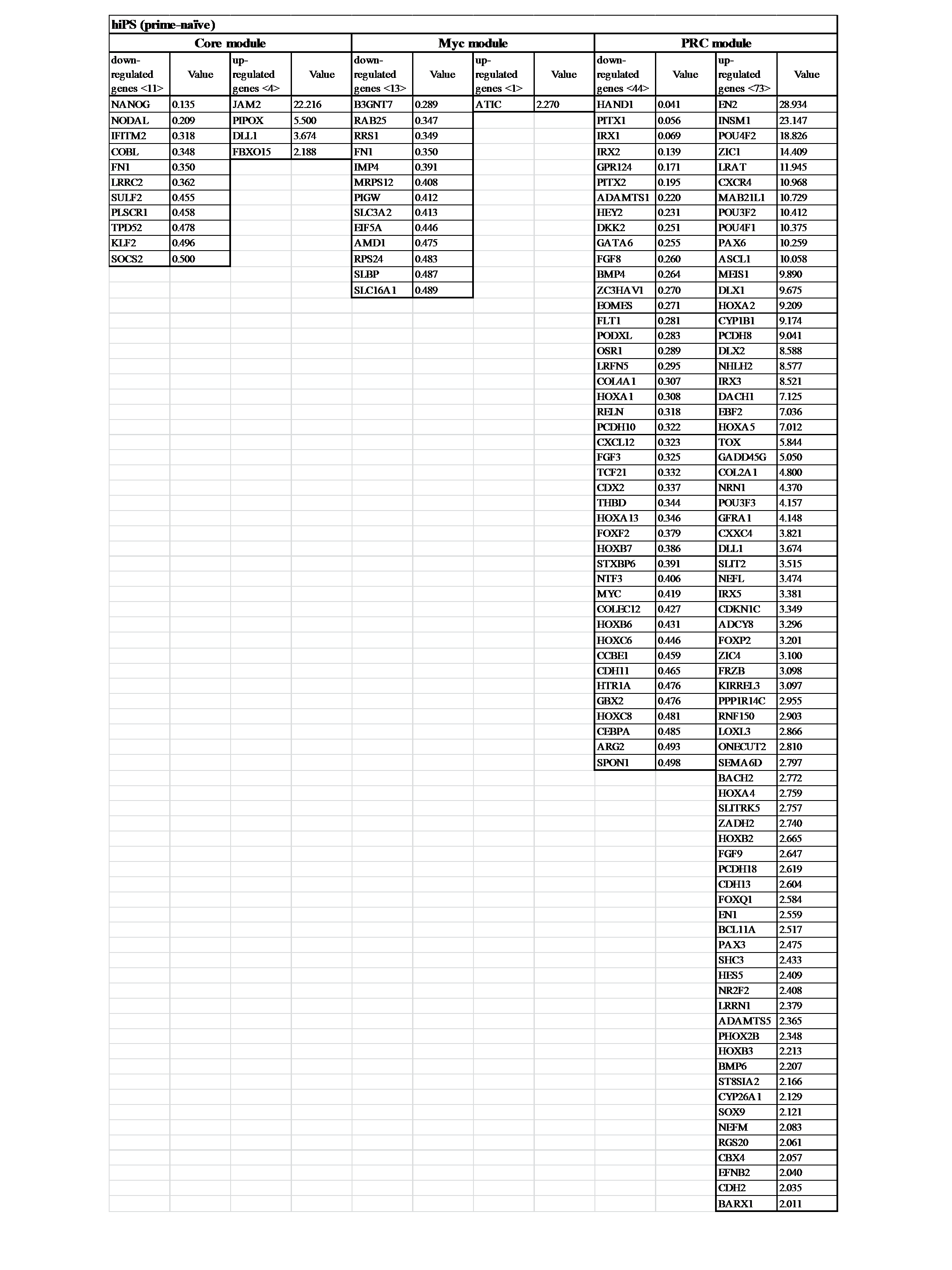

Supplement: Table S5 — Lists of Core, Myc and PRC module genes which show differential expression levels between human iPSCs in primed state and those in naïve state. (TIF) [file pone.0083769.s014.tif]

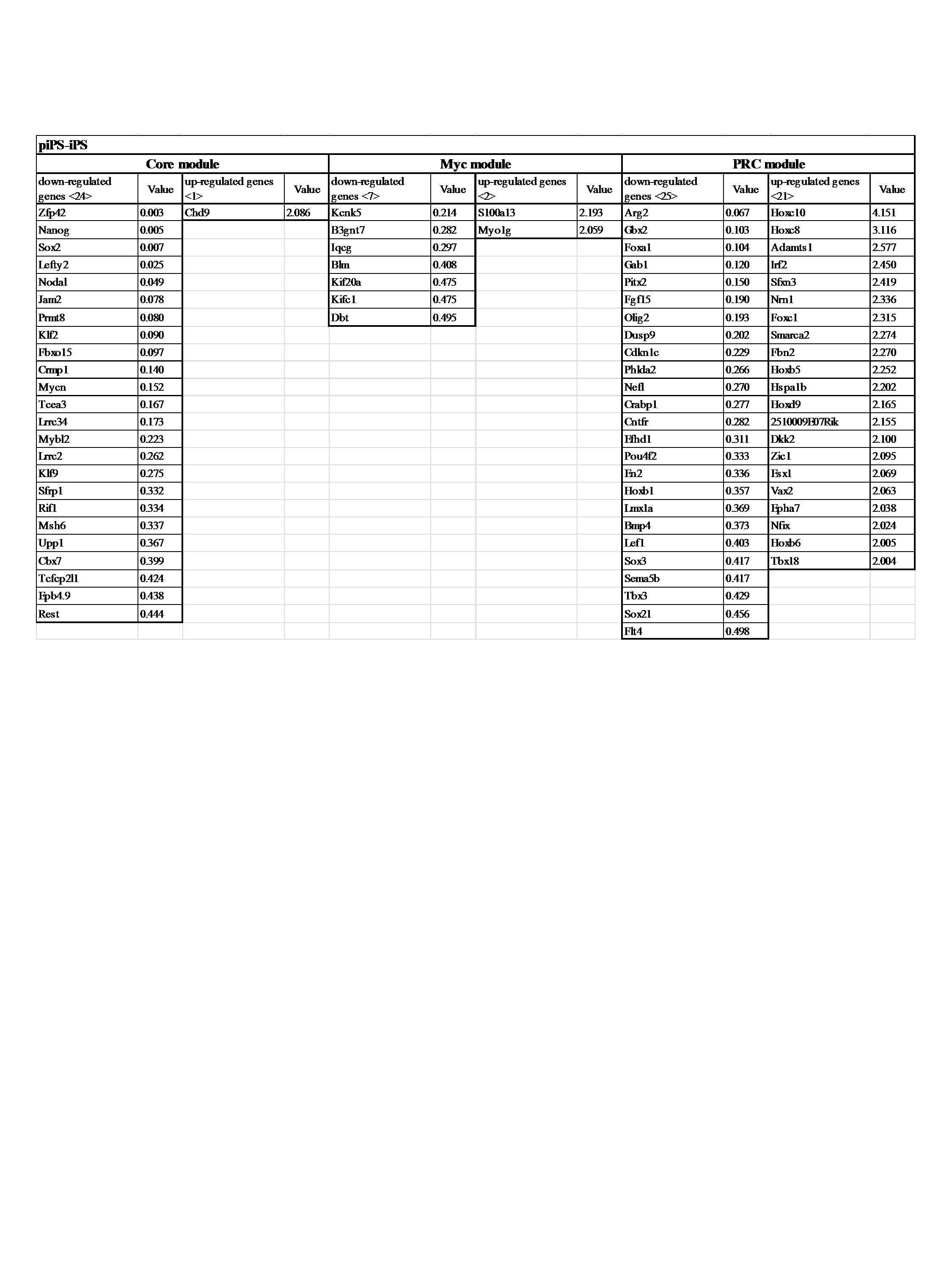

Supplement: Table S6 — Lists of Core, Myc and PRC module genes which show differential expression levels between partial and genuine iPSCs. (TIF) [file pone.0083769.s015.tif]
